# 3D Imaging of Axons in Transparent Spinal Cords from Rodents and Nonhuman Primates^1^,^2^,^3^

**DOI:** 10.1523/ENEURO.0001-15.2015

**Published:** 2015-04-08

**Authors:** Cynthia Soderblom, Do-Hun Lee, Abdul Dawood, Melissa Carballosa, Andrea Jimena Santamaria, Francisco D. Benavides, Stanislava Jergova, Robert M. Grumbles, Christine K. Thomas, Kevin K. Park, James David Guest, Vance P. Lemmon, Jae K. Lee, Pantelis Tsoulfas

**Affiliations:** Miami Project to Cure Paralysis, Department of Neurological Surgery, University of Miami School of Medicine, Miami, Florida 33136

**Keywords:** 3DISCO, axon regeneration, spinal cord injury, tissue clearing

## Abstract

The histological assessment of spinal cord tissue in three dimensions has previously been very time consuming and prone to errors of interpretation. Advances in tissue clearing have significantly improved visualization of fluorescently labelled axons. While recent proof-of-concept studies have been performed with transgenic mice in which axons were prelabeled with GFP, investigating axonal regeneration requires stringent axonal tracing methods as well as the use of animal models in which transgenic axonal labeling is not available. Using rodent models of spinal cord injury, we labeled axon tracts of interest using both adeno-associated virus and chemical tracers and performed tetrahydrofuran-based tissue clearing to image multiple axon types in spinal cords using light sheet and confocal microscopy. Using this approach, we investigated the relationships between axons and scar-forming cells at the injury site as well as connections between sensory axons and motor pools in the spinal cord. In addition, we used these methods to trace axons in nonhuman primates. This reproducible and adaptable virus-based approach can be combined with transgenic mice or with chemical-based tract-tracing methods, providing scientists with flexibility in obtaining axonal trajectory information from transparent tissue.

## Abstract (precis)

Recent advances in tissue clearing techniques have provided a promising method of visualizing axonal trajectories with unprecedented accuracy and speed. While previous studies have utilized transgenic labeling in mice, the use of virus or chemical neuronal tracers will provide additional spatiotemporal control as well as the ability to use animal models in which transgenic axonal labeling is not available.

## Significance Statement

Recent advances in tissue clearing techniques have provided a promising method of visualizing axonal trajectories with unprecedented accuracy and speed. While previous studies have utilized transgenic labeling in mice, the use of virus or chemical neuronal tracers will provide additional spatiotemporal control as well as the ability to use animal models in which transgenic axonal labeling is not available. We used adeno-associated viruses (AAVs) and chemical tracers and performed tetrahydrofuran-based tissue clearing to image multiple axon types in the rodent and nonhuman primate spinal cord using light sheet and confocal microscopy. This approach will provide scientists with a simple and flexible method of obtaining axonal trajectory information from transparent tissue.

## Introduction

Since Cajal’s successful use of Golgi’s stain to describe the structure of cells within the bird CNS (Cajal, 1888), increasingly accurate visualization of neural cells has been essential to progress in neuroscience. The use of anterograde and retrograde dyes as well as genetic labeling techniques has advanced our understanding of the structure−function relationship of the CNS. Most of this data is collected from two-dimensional histological sections, which makes it difficult to accurately study complex anatomical and structural changes that occur during development and disease. Therefore, three-dimensional visualization and quantification directly from whole CNS tissue would immensely benefit studies of CNS structure and function.

The use of three-dimensional visualization is especially important when quantifying new axonal growth after spinal cord injury (SCI). The use of histological sections is both time consuming and open to misinterpretation, since serial two-dimensional sections do not always provide accurate reconstructions of a three-dimensional organ (Luo et al., 2013). Axon fragments present in histological sections might be considered to have regenerated when in fact they were spared at the time of injury or sprouted from a spared axon. Such spared axons would be readily apparent in intact tissue since their origin can be traced back to regions rostral to the injury site. Intact pieces of lesioned mouse spinal cord have previously been cleared and imaged to demonstrate increased axonal regeneration following a conditioning sciatic nerve injury (Ertürk et al., 2012b), but these GFP-M mice had their axons prelabeled in a subset of neurons (Feng et al., 2000) in which all tracts were bilaterally labeled. A more tract-specific method to unilaterally trace regenerating axons requires the use of chemical-based tracers or fluorescent protein-expressing viral vectors such as adeno-associated virus (AAV). Importantly, these tracers and viruses can be used to study axons in a variety of different animal models in which transgenic fluorescent labeling is not readily available.

AAV2 is the most widely used serotype and has the highest selectivity for neurons (Tenenbaum et al., 2004). AAV8 has been reported to have a slight tropism for oligodendrocytes (Hutson et al., 2012), but has been shown to transduce rubrospinal tract (RST) and corticospinal tract (CST) fibers in adult rats better than AAV2 (Blits et al., 2010; Hutson et al., 2012). In addition to serotypes, another important consideration for viral vector-mediated gene expression is the strength of the promoter used for the gene-of-interest. The cytomegalovirus (CMV) promoter is commonly used, but other promoters, such as ubiquitin C (UbC), have also been utilized (Fitzsimons et al., 2002; Papadakis et al., 2004). Furthermore, since most transgenic reporter mice express green fluorescent protein (GFP), it might be desirable to use a viral vector that expresses red fluorescent protein.

Our goal in this study was to determine optimized methods for visualizing traced axons in cleared spinal cords from transgenic mice as well as nontransgenic animals. We compared different AAV serotypes, promoters, and fluorescent protein reporters and determined that AAV8 serotype with UbC promoter expressing GFP provides the strongest fluorescence signal after tetrahydrofuran-based tissue clearing, referred to as 3D imaging of solvent-cleared organs (3DISCO; Ertürk et al., 2012a). This viral tract-tracing method can be used to obtain convincing origin-target information for corticospinal and rubrospinal axons in the spinal cord in the mouse, rat, and nonhuman primates. This method is compatible with both light sheet fluorescent microscopy (LSFM), which can be used to image large segments of spinal cord with speed, as well confocal microscopes (such as point-scanning laser microscopes), which can provide much better resolution. In addition, this tract-tracing approach can be combined with transgenic mice in which different cell types are fluorescently labeled in order to study the relationship between axons and various cell types in the CNS. These methods provide researchers with a relatively easy, reproducible, and adaptable approach to investigate axon trajectories in intact CNS tissue with increased accuracy and speed.

## Materials and Methods

### Animals

Transgenic mice expressing GFP under the collagen1α1 promoter (*Col1α1-GFP* mice) were kindly donated by Dr. David Brenner (University of California San Diego, La Jolla, CA) (Yata et al., 2003). *GFAP-CreER* transgenic mice were obtained from The Jackson Laboratory (stock 012849, http://jaxmice.jax.org/strain/012849.html; Ganat et al., 2006). *Rosa26-tdTomato* reporter mice were kindly donated by Dr. Fan Wang (Duke University, Durham, NC) (Arenkiel et al., 2011). All mice were in a C57BL/6 genetic background. To generate *GFAPCreER-tdTomato* mice, *GFAP-CreER* and *Rosa26-tdTomato* mice were bred to each other to produce *GFAP-CreER^+^/Rosa26-tdTomato^fl/+^* or *^fl/fl^* offspring. *GFAPCreER-tdTomato* mice (6-7 weeks old) received tamoxifen (50 mg/ml diluted in 9:1 sunflower oil:ethanol) daily for 5 consecutive days (0.125 mg/g, i.p.). One week after the last injection, mice received SCI as described below. Adult female Fischer rats (180-200 g; Harlan) and one 5-year-old male, nonhuman primate (*Macaca fascicularis*, The Mannheimer Foundation) were also used in this study. All animal procedures were performed in accordance with the University of Miami and NIH animal care and use guidelines.

### Plasmids and virus production

We used an AAV vector that expressed eGFP under the human cytomegalovirus immediate-early promoter and another AAV vector that expressed eGFP or tdTomato under the human ubiquitin C promoter (Chung et al., 2006). The plasmids and the packaging plasmid AAV8 733 (Petrs-Silva et al., 2009) and pHelper (Agilent Technologies) were cotransfected into 293T cells at 70% confluence using the calcium phosphate precipitation method. The cells were incubated for 48 h at 37 °C and 5% CO_2_. After 48 h, the cells were collected and freeze-thawed three times to release the AAV particles from the cells. After 30 min of Benzonase Nuclease (Sigma) treatment, the crude lysate was clarified by low-speed centrifugation. The supernatant was loaded on discontinuous iodixanol step gradients (Zolotukhin et al., 2002) in OptiSeal tubes (Beckman Coulter) and centrifuged in a Type 70 Ti rotor (Beckman Coulter) at 69,000 rpm (350,000 g) for 1 h at 18 °C. The AAV particle-containing fraction was collected and further purified using an AKTA FPLC system (GE Healthcare) by column chromatography on a 5 ml HiTrap column (GE Healthcare). About 25 ml was eluted from the column using elution buffer (20 mM Tris, 215 mM NaCl, pH 8.0) and then the AAV particles were concentrated and buffer exchanged to 200 μl in HBSS (Invitrogen) using an Amicon Ultra-15 50K concentrator (Millipore). The purified AAV particles were then titered for genome content using real-time qPCR. Titers were in the range of 1-3 × 10^14^ GC (Genome Copy) per milliliter. The three AAV vectors with the UbC promoter (AAV-UbC, AAV-UbC-eGFP, AAV-UbC-tdTomato) have been deposited at Addgene.

### SCI

#### Mice

Seven- to 9-week-old female mice were anesthetized (ketamine/xylazine, 100/15 mg/kg, i.p.) and received mid-thoracic (T8) laminectomies before undergoing spinal cord injuries. For dorsal hemisections, the dura was punctured with a 30 G needle above the dorsal horns, and a superfine iridectomy scissor was used to cut the dorsal half of the cord at a depth of 0.8 mm. A Micro Feather ophthalmic scalpel was used to retrace the lesion to ensure its completeness. For contusion injury, the spinal column was stabilized using a spinal clamp and the exposed T8 cord placed below the impacter of an Infinite Horizon Impactor device (Precision Systems and Instrumentation, 75 kDynes). Injured mice received lactated Ringer's solution, antibiotics (Baytril, 10 mg/kg), and analgesics (buprenorphine, 0.05 mg/kg) subcutaneously for the first week after surgery. Twice daily bladder expressions continued for the duration of the study.

#### Rats

Ten days after the injection of viral particles (see below), Fischer rats were anesthetized (ketamine/xylazine, 80/10 mg/kg, i.p.) and received moderate contusion at T8 as described above for mice (Infinite Horizon Impactor at 200 kD). Injured rats received lactated Ringer's solution (6 ml), antibiotics (Gentamycin, 0.05 mg/kg), and analgesics (buprenorphine, 0.01 mg/kg) subcutaneously twice daily for the first week after surgery. Twice daily bladder expressions continued for the duration of the study. In a separate study, Fisher rats were anesthetized as above and a laminectomy performed from T13 to L2 to expose the L4-L6 spinal cord. Contusion of the left L4-L6 spinal cord was induced using a NYU impact device (10 g × 12.5 mm; Gruner, 1992). This SCI targets the lateral and medial gastrocnemius motor pool. This study meets the standards of the Minimum Information About a Spinal Cord Injury Experiment (MIASCI, Lemmon et al., 2014). An excel file containing the information has been uploaded onto RegenBase (www.regenbase.org).

### Tracer injections

#### Mice

Immediately after SCI, mice were placed on a stereotaxic frame (Stoelting). To label the CST, a craniotomy was performed to expose the right sensorimotor cortex and AAV virus was injected into the cortex using a nanoliter injector (WPI) attached to a pulled glass pipette. Six injections (200 nl per site) were performed at a rate of 50 nl/min at the following coordinates: 1.0 mm and 1.4 mm lateral; 0.7 mm deep; 0.1 mm, 0.6 mm, and 1.1 mm posterior to bregma. For rubrospinal tract tracing, AAV (500 nl) was injected at a rate of 50 nl/min at 0.6 mm lateral, 3.7 mm deep, and 3.2 mm posterior to bregma. The needle was left in place for 1 min before moving to the next site.

#### Rats

For labeling the CST, rats anaesthetized by ketamine/xylazine (80/10 mg/kg) were placed on a stereotaxic apparatus connected to a QSI injector kit (Stoelting). A craniotomy was made over the left sensorimotor cortex and virus was injected at two sites (1 μl each at a rate of 0.1 μl/min): 1.2 mm and 2 mm lateral; 0.7 mm deep; 1.7 mm posterior to bregma. For labeling both CSTs, this procedure was repeated on the right sensorimotor cortex. For labeling the dorsal root ganglia (DRG) sensory tracts, rats were anesthetized as described above and a midline skin incision was made at the occipital protuberance (1-2 cm in length). An incision through the trapezius muscle was made and the underlying muscles were blunt dissected and retracted from their attachment to the dorsal skull to expose the atlanto-occipital cisternal membrane. The virus was then injected 100 μm below the punctured membrane at a rate of 0.4 μl/min and a total volume of 2 μl.

#### Nonhuman primate

Macaque was fasted 12 h prior to the procedure. Anesthetic induction used a combination of ketamine/xylazine (5/0.25 mg/kg) for endotracheal intubation, followed by maintenance with isoflurane (1.5% and 2% oxygen). After the airway was secured, the animal was positioned prone on a stereotactic frame (KOPF Instruments) and the head was fixed in position. A craniotomy was drilled starting on the midline 1 cm rostral to the coronal suture, extending its borders diagonally following the direction of the suture 3 cm laterally on each side, then caudally on a parasagittal line 3 cm on each side and returning diagonally towards the midline 2 cm behind the coronal suture completing the flap. The bone flap was carefully removed and the dura opened with a #11 blade to expose the cortex. A motorized stereotaxic injector (Stoelting) was mounted on the frame, and a 10 µl Hamilton syringe with a 32 G needle was used to inject the right primary motor cortex with fluoro-Ruby (150 nl per site, D1817; Invitrogen). After tracing, the dural edges were approximated and sutured in a water-tight closure using 6-0 prolene. Burr holes were drilled in the cranial flap, which was secured in position with sutures. The incision was then closed by layers and spontaneous extubation was allowed. The animal was monitored in an ICU unit for the following week and then transferred to its regular room. Prophylactic antibiotic (ceftriaxone) was given preoperatively (1 g) and postoperatively (1 g) for 3 d. Analgesics were given for 5 d postoperatively (buprenex 0.01 mg/kg BID and meloxicam 0.3 mg/kg/d). After tracer injection, the animal survived for 10 weeks before euthanasia and transcardial perfusion with 4% paraformaldehyde (PFA).

#### Retrograde labeling of motoneurons

Cholera toxin B subunit (CTB; C-22843; Invitrogen) was dissolved in 0.9% saline to a final concentration of 1%. After anesthetizing with 1.5% isofluorane and 2% oxygen, each medial and lateral gastrocnemius (MG and LG) muscle was carefully dissected in order to avoid damage to the blood supply or nerves, and the muscle fasciae were left intact. CTB (5 µl per muscle) was injected at three sites in the proximal part of each muscle head, near where the nerve entered the muscle, using a Hamilton microsyringe. One week later, rats were anesthetized and transcardially perfused with 4% PFA and the spinal cords dissected.

### Quantifications

#### Comparing labeling between different AAVs

**Figure 1:**
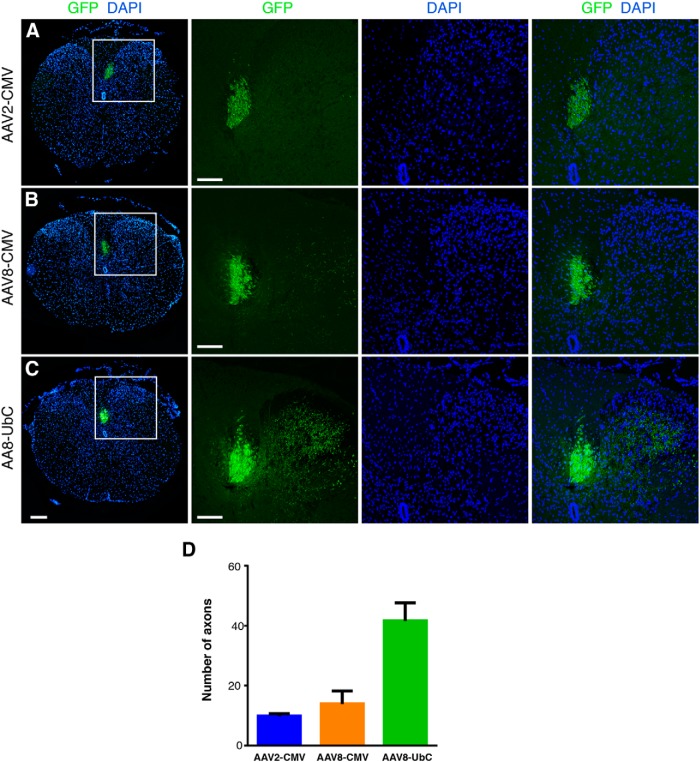
Comparison of AAV-GFP labeling of the mouse corticospinal tract. ***A***, ***B***, AAV8-CMV-GFP (***B***) labeled the dorsal CST slightly more efficiently than AAV2-CMV-GFP (***A***). ***C***, However, AAV8-UbC-GFP labeled the dorsal tract and collaterals most efficiently compared with the other two viruses. DAPI-labeled nuclei are in blue. Insets show higher magnification of the dorsal CST and their collaterals. All images are from coronal tissue sections of mouse thoracic spinal cord 2 weeks postinjection. Scale bars: leftmost panels, 200 μm; all other panels, 100 μm. ***D***, Comparison of number of axons labeled in the gray matter between different viruses. Axons quantified from specific regions of interest as detailed in Materials and Methods. Error bars represent SEM. *n* = 2 biological replicates per group.

**Figure 2:**
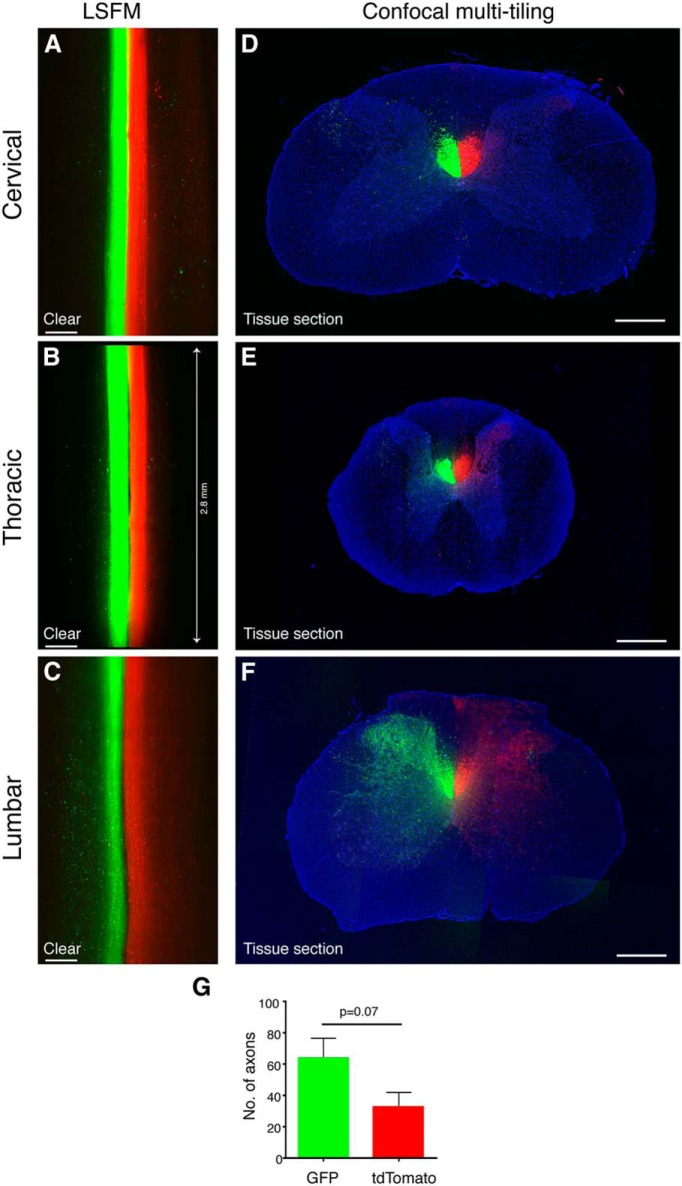
Comparison of AAV8-UbC-GFP and AAV8-UbC-tdTomato labeling of the rat corticospinal tract. ***A***, ***B***, ***C***, Horizontal optical sections of cleared spinal cords from different levels imaged with LSFM. ***D***, ***E***, ***F***, Confocal images of coronal tissue sections of spinal cord regions adjacent to ***A***, ***B***, and ***C***, respectively. ***G***, Quantification of axon collaterals in the gray matter from tissue sections show greater labeling with GFP than tdTomato. Axon quantified from specific regions of interest as detailed in Materials and Methods. Line in ***B*** shows the length of the cleared cord. All images are from rat spinal cord at 8 weeks after AAV injection. Error bars represent SEM. *p* = 0.07 using unpaired two-tailed Student’s *t* test. *n* = 3 biological replicates. Scale bars: ***A−C***, 300 μm; ***D−F***, 500 μm.

Axons were manually counted in cervical gray matter tissue sections with ImageJ software. To count the axons in the gray matter contralateral to the labeled main CST, three vertical rectangular regions of interest (ROI; 10 μm × 1300 μm) were drawn (1) adjacent, (2) 500 μm, and (3) 1000 μm from the central canal. CST axons detected in each ROI were manually counted for each tissue section. Three cervical spinal cord sections were counted for each animal. The average number of CST axons in each ROI across the three sections were averaged together to obtain the final number of CST axons per mouse. This method was performed for quantifications in Figures 1*D* and 2*G*.

#### Axonal tracing and counting using Imaris

Using the FilamentTracer module, fibers within a selected ROI were detected with semimanual segmentation. Briefly, the fiber is traced in the *x*−*y* position while Imaris simultaneously determines the *z*-position placement. This method was performed for quantifications in Figure 5I.

#### Motoneuron counting using Imaris

After manually selecting the regions containing the two motoneuron columns, Imaris Spots function was used to automatically detect CTB-labeled motoneurons. After automatic detection, regions were manually inspected and corrected for any false-positive or false-negative detections.

#### Analyses of VGLUT1 and GFP positive synaptic contacts

Frozen sections of rat lumbar spinal cords with labeled sensory axons were immunostained with antibodies against NeuN (dilution 1:250; #MAB377, Millipore), vesicular glutamate transporter 1 (vGlut1; dilution 1:500; #135 303, Synaptic Systems), vGlut2 (dilution 1:2000; #AB2251, Millipore), SV2 (dilution 1:400; DHSB), and GAD-6 (dilution 1:500; DHSB). NeuN immunopositive cells in lamina IX of the spinal cord were imaged using confocal microscopy. Twelve motoneurons from three different animals were randomly sampled. Thirty to forty optical *z*-sections, every 0.25 μm, were imaged for each field. The cellular perimeter of NeuN positive cell bodies and the origin of primary dendrites were rendered in three dimensions (3D) using the surface tool of the Imaris software. The number of immunofluorescent varicosities juxtaposed to the 3D surface was measured using the surface and spot tools of the Imaris software. An average density estimate was obtained for each cell. We took extra care to ensure that the vGlut1^+^/GFP^+^ fiber endings were in contact with the surface of the neuron by viewing the *z*-sections in the orthogonal view and rotating the sample at different angles to avoid counting processes and varicosities that were in separate planes.

### Tissue clearing and microscopy

After transcardial perfusion with 4% PFA as described above, spinal cords were postfixed in 4% PFA and then washed in PBS (both overnight at 4 °C). The dura was carefully and completely removed as residual dura can trap bubbles that prevent effective LSFM. Then the spinal cord was cut into ∼8-mm-long segments (centered at T8 or L5 for the injury site). Samples were incubated (on a rotating shaker at room temperature) in 50%, 80%, and 100% peroxide-free THF (Sigma 401757), each for 2 h for mice, 2.5 h for rats, and 3.5 h for macaque and then 100% THF overnight. Peroxides were removed from THF by passing 100% THF through a chromatography column filled with basic activated aluminum oxide (Sigma 199443) as previously described (Becker et al., 2012). (Warning: this process also removes the stabilizer from THF, which may explode after prolonged exposure to oxygen or sunlight. Thus, users must add 250 mg/l butylated hydroxytoluene (Sigma W218405) to THF after peroxide removal.) The next day, samples were transferred to BABB solution (1:2 ratio of benzyl alcohol, Sigma, 305197; and benzyl benzoate, Sigma, B6630) for 2.5-3 h. After clearing, samples were immediately imaged by LSFM (Ultramicroscope, LaVision BioTec). The ultramicroscope uses a fluorescence macro zoom microscope (Olympus MVX10) with a 2× Plan Apochromatic zoom objective (NA 0.50) and a total magnification of 12.5×. For confocal microscopy, smaller (300-400 μm) cross-sections were cut from the original 8-mm-long segments and imaged using an Olympus FV1000 Fluoview confocal microscope using the 10× (NA 0.4), 40× (NA 1.3), and 60× (NA 1.42) objectives. Image analysis and 3D reconstructions were performed using Imaris v7.7.2 software (Bitplane) after removing autofluoresence using the Imaris Background Subtraction function with the default filter width so that only broad intensity variations were eliminated. Additionally, the entire spinal cord was defined as an ROI in order to mask all background fluorescence outside the spinal cord surface. Artifact and nonspecific fluorescence surrounding the spinal cord was segmented and removed using the automatic isosurface creation wizard based upon absolute intensity. Voxels contained within the created surface were set to zero and the remaining mask was used for all further analysis. Automatic segmentation of neurons within specified ROIs was applied using the spots detection function and later superimposed on a maximum intensity projection volume rendering of the tissue. Quality thresholds were set based upon visual inspection of the mixed model rendering. Movies were generated using Imaris and merged in Photoshop CS6.

## Results

### Comparison of AAV serotypes, promoters, and fluorescent proteins for optimal axon labeling

Since THF-based tissue clearing can quench fluorescent signals, achieving strong fluorescent labeling is critical to obtain quality images. Thus, our first goal was to determine the optimal combination of AAV serotype and promoter for robustly labeling specific supraspinal tracts. Several studies have shown that the transduction efficiency and tropism of CNS and PNS neurons can vary considerably (Chamberlin et al., 1998; Taymans et al., 2007; Cearley et al., 2008; Ayuso et al., 2010; Mason et al., 2010). Furthermore, the level of expression of reporter genes such as *GFP* depends on the promoter used by these recombinant viruses (Paterna et al., 2000; Paterna and Büeler, 2002). Therefore, to identify the best virus serotype/promoter combination that labels various axonal tracts with strong fluorescence, we used AAV2 and AAV8 expressing *GFP* under the control of either the human CMV or the human UbC promoters.

We injected the viruses in the motor cortex of adult mice to transduce layer V pyramidal neurons and to label CST axons. Two weeks postinjection, we analyzed the labeling intensity of the CST in histological sections at the thoracic levels between T6 and T10. While both AAV2 and AAV8 serotypes with the CMV promoter expressed GFP in CST axons in the dorsal funiculus with very few collateral axons labeled in the gray matter, AAV8 labeled a slight greater number of axons than AAV2 (Fig. 1). Next, we compared the number of GFP-labeled axons under the UbC and CMV promoters using AAV8, and determined that fine collaterals are better visualized with the UbC promoter (Fig. 1). In fact, selection of the UbC promoter over the CMV promoter had a stronger effect on GFP labeling than selection of AAV8 over AAV2. Based on our results, we decided to use the AAV2- or AAV8-UbC-GFP virus for axon labeling in most of the ensuing experiments.

Next, we compared the labeling efficiencies of GFP and tdTomato fluorescent proteins and their compatibility with tetrahydrofuran-based tissue clearing by injecting AAV8-UbC-GFP or AAV8-UbC-tdTomato into each motor cortex to label each CST with different fluorescent proteins in the same rat. At 8 weeks after virus injection, segments of the cervical, thoracic, and lumbar spinal cord were rendered transparent using a modified 3DISCO method and adjacent segments were also processed for tissue sectioning. Modifications to the 3DISCO method included exclusion of dichlorormethane, different THF catalog numbers, and longer incubation times (see Materials and Methods for details). Images of the cleared spinal cord obtained with LSFM demonstrated that while the CST was labeled with similar fluorescent intensities along the entire length of the spinal cord by both fluorescent proteins (Fig. 2*A−C*), there were more collaterals visible with GFP than with tdTomato (Fig. 2*C*). Since this could be due to differences in labeling efficiency and/or susceptibility to fluorescence quenching by the tissue clearing method, we used tissue sections (rather than cleared tissue) from adjacent regions to compare GFP and tdTomato labeling. Confocal images of tissue sections demonstrated that GFP labeled about twice as many CST collaterals in the gray matter compared to tdTomato (Fig. 2*D−G*). This suggested that while potential differences in fluorescence quenching cannot be ruled out, the greater number of GFP-labeled axons visualized after tissue clearing was due, at least in part, to greater labeling efficiency by AAV8-UbC-GFP than by AAV8-UbC-tdTomato. Therefore, while both GFP and tdTomato can be used to label supraspinal axons, GFP may provide better axonal labeling than tdTomato.

### Determining axon trajectory in cleared tissue using tract-tracing

**Figure 3:**
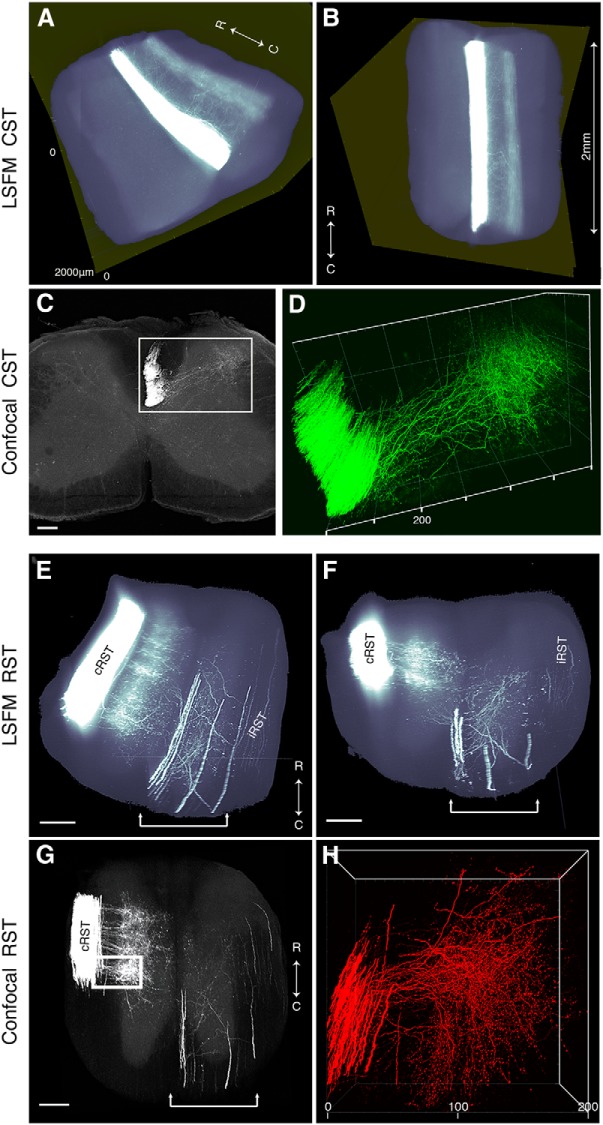
CST and RST axons can be visualized in cleared mouse spinal cord using AAVs. ***A***, 3D reconstruction from serial LSFM optical sections of a segment of the upper thoracic spinal cord from a mouse injected with AAV8-UbC-GFP into sensorimotor cortex to label CST axons (white; see also Fig. 1). ***B***, Dorsal view of the same 3D reconstruction as in ***A***. ***C***, ***D***, Confocal microscopy, which has higher resolution than LSFM, can better distinguish individual CST axons (see also Movie 1). Boxed area in ***C*** is represented in ***D***. ***E***, 3D reconstruction from serial LSFM optical sections of a cleared segment of the upper thoracic spinal cord from a mouse injected with AAV2-UbC-tdTomato into the red nucleus to label RST axons (white). ***F***, Coronal view of the same 3D reconstruction as in ***A***. ***G***, Projection view of coronal image stack taken with confocal microscopy from cleared spinal cord (as shown in ***E*** and ***F***). Bracketed regions in ***E***, ***F***, and ***G*** refer to non-RST axons that were mislabeled during virus injection. Boxed area in ***G*** is shown at higher magnification in ***H*** (see also Movie 2). All images are taken from cleared mouse midthoracic spinal cord tissue at 4 weeks after virus injection. Scale bars: ***C***, 80 μm; ***E−G***, 100 μm. *n* = 3 biological replicates per group. iRST, Ipsilateral RST; cRST, contralateral RST.

Using this modified 3DISCO method for rendering the CNS tissue transparent, we set out to examine two specific supraspinal pathways, the CST and RST, in the injured spinal cord. Because of their important role in motor control as well as their presence as distinct bundles in the spinal cord, both tracts have been the focus of investigation in multiple regeneration studies. Four weeks after moderate contusive T8 SCI and injection of AAV8-UbC-GFP into the motor cortex of adult mice, we used LSFM to optically section 8-mm-long cleared spinal cord at the lower cervical level. We were able to scan the entire piece of the spinal cord (∼1 mm thick, 400-500 optical sections) in less than 3 min, with the main component of the CST appearing as a single fluorescent bundle in the dorsal funiculus and collateral fibers extending to the dorsal gray (Fig. 3*A*,*B*, 2 mm segment shown). The resolution was sufficient to resolve some single collateral branches and axons traveling in the dorsolateral CST (Fig. 3*A*, Movie 1), but insufficient to resolve most individual axons within the main CST and gray matter (Movie 1). Therefore, we cut this same piece of spinal cord (as shown in Fig. 3*A*,*B*) into 300- to 400-μm-thick transverse sections, and placed them on a cover slip to image with confocal microscopy. With a 10× dry objective, we show that the main CST and its collaterals can be partially resolved (Fig. 3*C*). With a 40× oil objective, it was possible to resolve single axons within the main CST bundle and follow the trajectory of their collateral branches all the way to their terminal boutons (Fig. 3*D*, Movie 1).

Movie 1 Composite 3D movie of the CST imaged with LSFM and confocal microscopy of mouse thoracic spinal cord rostral to the injury site. Magenta axons in the confocal segment of the movie represent axons that were traced using the Imaris software. Corresponds to Figure 3*A−D*. 10.1523/ENEURO.0001-15.2015.video.1

Next, we traced the RST by injecting AAV2-UbC-tdTomato into the red nucleus immediately after a T8 moderate contusive SCI (mice euthanized 4 weeks later). Since RST axons are thicker than CST axons, LSFM had sufficient resolution to image axons in RST tracts that were observed clearly in the lateral funiculus (Fig. 3*E*,*F*, Movie 2), with collaterals spreading into laminae 5, 6, and the dorsal portion of 7 (Liang et al., 2012). Using confocal microscopy, we imaged a region more caudal to that shown in Figure 3*E* and observed that axon collaterals and terminal boutons could be visualized more clearly (Fig. 3*G*,*H*, Movie 2).

Movie 2 Composite 3D movie of the RST imaged with LSFM and confocal microscopy of mouse thoracic spinal cord rostral to the injury site. Corresponds to Figure 3*E−H*. 10.1523/ENEURO.0001-15.2015.video.2

Injected virus/tracers can often diffuse into other surrounding nuclei that lie close to the red nucleus, and these mislabeled axons are often misinterpreted as regenerating axons using traditional histological sectioning methods (Steward et al., 2003). In fact, in our study, we occasionally observed axons traveling in the ipsilateral ventromedial funiculus innervating laminae 7, 8, and 9 (bracketed region in Fig. 3*E−G*). However, using 3D reconstructions from cleared spinal cords, these mislabeled axons were clearly visible in regions rostral to the injury site and were easily distinguishable from the actual rubrospinal tracts.

Our results demonstrate that supraspinal tracts traced with AAV2 or AAV8 expressing GFP or tdTomato under the UbC promoter can be visualized using LSFM or confocal microscopy using THF/BABB-based tissue clearing methods. LSFM provides a very rapid method of imaging large diameter axons (e.g., RST axons) in whole spinal cord tissue, and can allow investigators to clearly identify mislabeled axons that can be mistaken as regenerating axons after injury. However, thin axons such as those of the CST may require confocal (or two-photon) microscopy to resolve fine collaterals and terminals that extend into the gray matter.

### Axonal organization in relation to the scar at the injury site

**Figure 4:**
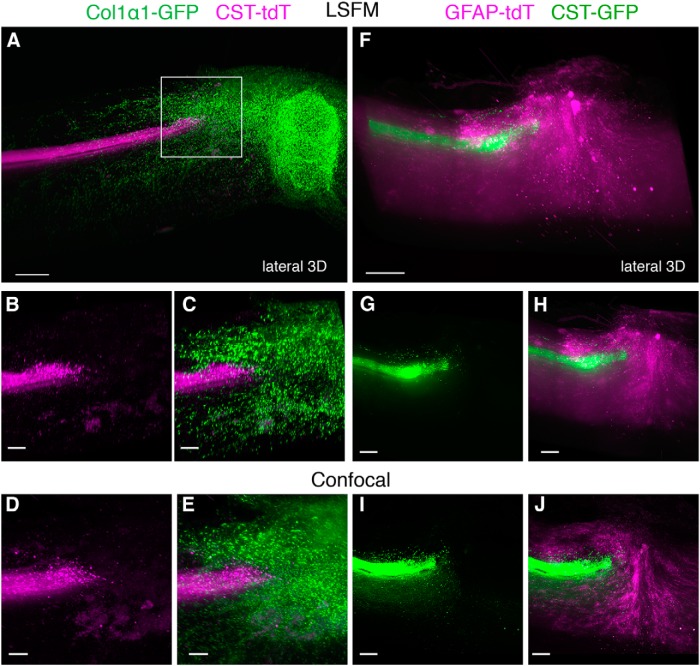
AAV-labeled CST axons in relation to the fibrotic and astroglial scar after SCI in mice. ***A−E***, AAV8-UbC-tdTomato was used to label CST axons in *Col1α1-GFP* mice where fibroblasts are labeled with GFP (*n* = 3 biological replicates) (see also Movie 3). ***A−C***, At 4 weeks after a contusive SCI, 3D reconstruction of the injury site using LSFM shows the lesioned CST (magenta) terminating at the rostral edge of the fibrotic scar (green). ***D***, ***E***, 3D reconstruction using confocal microscopy shows greater number of CST axons and fibroblasts. ***F−J***, AAV8-UbC-GFP was used to label CST axons in *GFAPCreER-tdTomato* mice where astrocytes are labeled with tdTomato (see also Movie 4). ***F−H***, At 4 weeks after a dorsal hemisection, 3D reconstruction of the injury site using LSFM showed lesioned CST axons (green) terminating around the astroglial scar (magenta). While the fluorescent signal was not sufficient to clearly identify the astroglial scar using LSFM, confocal microscopy provided much better results (***I***, ***J***, *n* = 3 biological replicates). All images are from cleared mouse spinal cord 4 weeks after midthoracic injury and virus injection. Boxed area in ***A*** is represented in ***B−E***. Scale bars: ***A***, ***F***, 200 μm; ***B−E***, 50 μm; ***G−J***, 100 μm.

**Figure 5:**
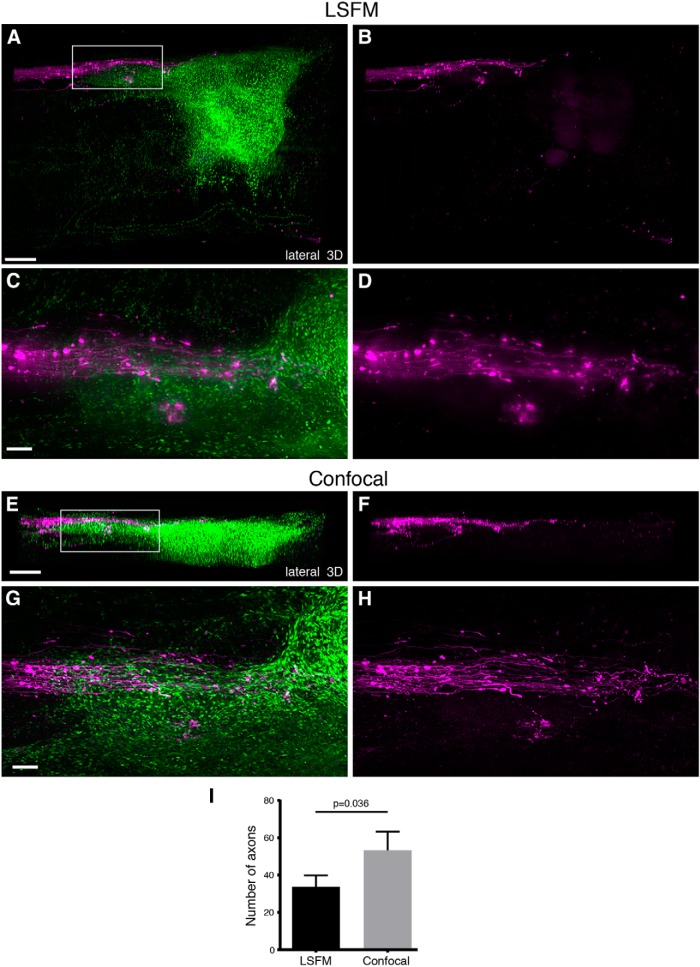
AAV-labeled RST axons in relation to the fibrotic scar after SCI in mice. 3D reconstruction of the injury site using LSFM (***A−D***) or confocal (***E−H***) microscopy after using AAV2-UbC-tdTomato to label the RST in *Col1α1-GFP* mice. Lesioned RST axons (magenta) terminate at the rostral edge of the fibrotic scar (green) at 4 weeks after contusive SCI. While confocal microscopy could not image through the entire depth of the spinal cord due to the limited working distance of the 10× objective (***A*** vs ***E***), the visualization of individual axons was much better (***D*** vs ***H***) than with LSFM, as indicated by the quantification of the number of axons that could be detected by the two imaging methods (***I***). The axons were counted from a region of interest that included the entire CST bundle in 3D as shown in ***D*** and ***H***. *p* = 0.036 using paired two-tailed Student’s *t* test. All images are from cleared mouse spinal cord injury site. Boxed area in ***A*** is represented in ***C*** and ***D***. Boxed area in ***E*** is represented in ***G*** and ***H***. Scale bars, 100 μm. *n* = 5 biological replicates. See also Movie 5.

When investigating axon regeneration, it is often useful or even necessary to obtain information about the spatial relationship between axons and scar tissue that forms at the injury site. However, since previous tissue-clearing studies have focused solely on axons, we sought to identify transgenic mouse models of scar formation that can be used with the axon tracing and tissue clearing methods described above. The same viruses as above were used to visualize axons in relation to markers of the fibrotic scar (Col1α1-GFP^+^ fibroblasts) or the astroglial scar (GFAPCreER-tdTomato^+^ astrocytes), and were imaged using both LSFM and confocal microscopy as described above. In *Col1α1-GFP* mice, the CST was labeled with AAV8-UbC-tdTomato and the main CST tract was observed as a single fluorescent bundle that abruptly ended at the contusion injury site filled with Col1α1-GFP^+^ fibroblasts (Fig. 4*A*, Movie 3). A region of interest centered at the rostral edge of the fibrotic scar showed severed axons failing to extend into the Col1α1-GFP^+^ region. LSFM was able to image through the entire depth of the injury site, while images taken using confocal microscopy with a 10× dry objective were limited to the dorsal portion of the injury site due to the limited working distance of the objective (Fig. 4*B−E*). RST axons were also observed terminating at the Col1α1-GFP^+^ fibrotic scar, and LSFM was sufficient to resolve individual axons (Fig. 5*A−D*, Movie 5), presumably due to their larger diameter. Nonetheless, confocal microscopy still provided better resolution that allowed for approximately 50% more axons that could be detected compared to LSFM (Fig. 5*I*).

Movie 3 3D LSFM movie of an injured midthoracic spinal cord of a transgenic mouse expressing GFP in fibroblasts, showing the lesioned CST labeled with AAV8-UbC-tdTomato and the fibrotic scar labeled with GFP. Corresponds to Figure 4*A*. 10.1523/ENEURO.0001-15.2015.video.3

Movie 4 3D LSFM movie of an injured midthoracic spinal cord of a transgenic mouse expressing tdTomato in astrocytes and the lesioned CST labeled with AAV8-UbC-GFP. Corresponds to Figure 4*F*. 10.1523/ENEURO.0001-15.2015.video.4

Movie 5 3D LSFM movie of an injured spinal cord of a transgenic mouse expressing GFP in fibroblasts showing the lesioned RST labeled with AAV8-UbC-tdTomato and the fibrotic scar labeled with the GFP. Corresponds to Figure 5*A−D*. 10.1523/ENEURO.0001-15.2015.video.5

To assess the astroglial scar, we bred *GFAP-CreER* mice with *Rosa26-tdTomato* reporter mice to generate *GFAPCreER-tdTomato* mice in which a small percentage of astrocytes are labeled with tdTomato. We used this strategy to demonstrate the feasibility of performing tissue clearing in *Rosa26* reporter mice, which are commonly used in genetic fate mapping studies. We used AAV8-UbC-GFP to label CST axons and performed T8 dorsal hemisection in *GFAPCreER-tdTomato* mice. We chose to use dorsal hemisection for these studies because contusive injuries resulted in significant accumulation of autofluorescent immune cells that interfered with accurate image acquisition (data not shown). The fluorescence intensity of astrocytes in *GFAPCreER-tdTomato* mice was not as strong as that observed in Col1α1-GFP^+^ fibroblasts, and we found it difficult to clearly delineate astroglial scar regions in 3D reconstructions from LSFM images (Fig. 4*F−H*, Movie 4). Confocal microscopy provided better resolution of individual axons and astrocytes (Fig. 4*I−J*), but was not able to image through the entire depth of the injury site. Our studies demonstrate that while *Col1α1-GFP* mice are compatible with the 3DISCO tissue clearing method to study axon−scar relationships in the injured whole spinal cord, more experiments are necessary to determine the utility of *Rosa26* reporter mice in similar studies.

### CST axon projections in the injured rat spinal cord

**Figure 6:**
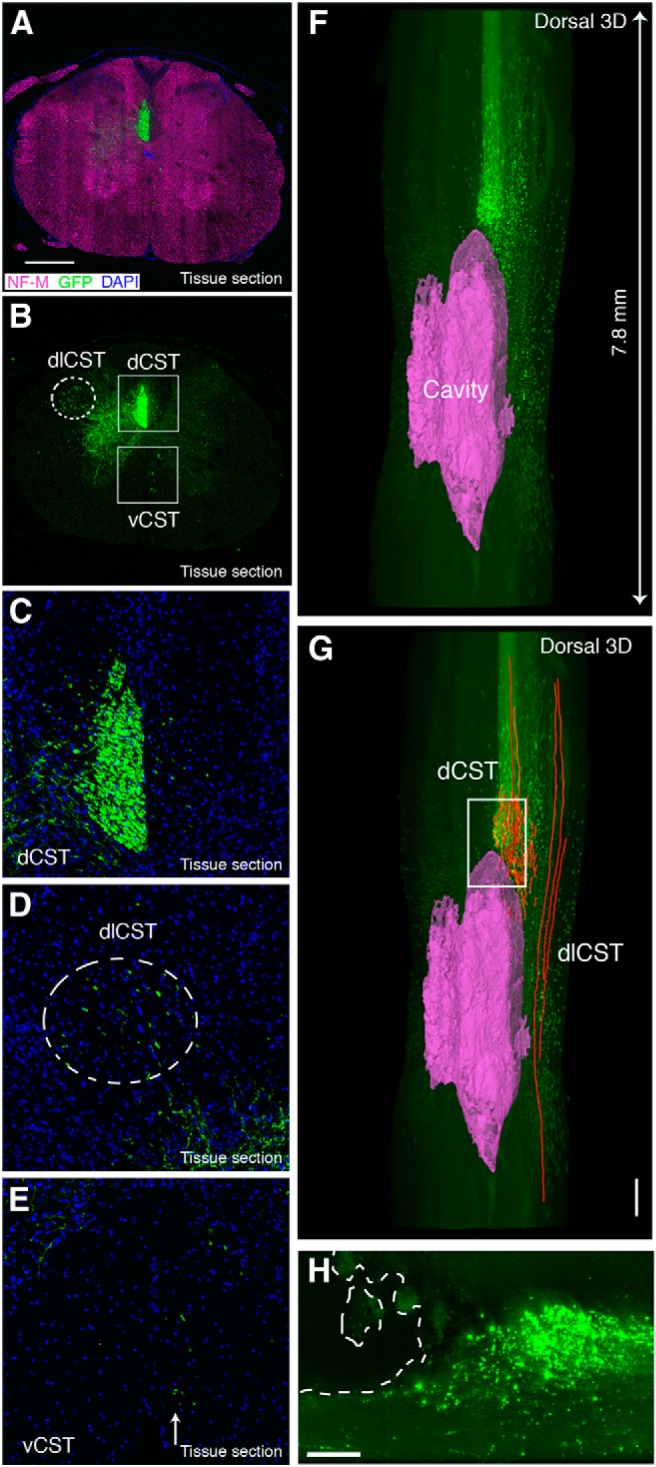
AAV-labeled CST axons in relation to the cavity after SCI in rats. ***A−E***, Confocal images of coronal tissue sections from the upper thoracic segments (rostral to the injury site) after using AAV8-UbC-GFP to label CST in rats. All CSTs including the main dorsal CST (dCST; ***B***, ***C***), the minor dorsolateral CST (dlCST; ***B***, ***D***), and the ventral CST (vCST; ***B***, ***E***) were labeled with this method. ***F−H***, 3D reconstruction of LSFM images from cleared injured spinal cord (dorsal view) showing the lesioned CST terminating at the rostral portion of the cavity (magenta) (see also Movie 6). Axon tracing (***G***, red lines) shows spared dlCST next to the cavity. Cavity reconstruction and axon tracing were performed using Imaris software. ***H*** is a maximum projection image of 15 LSFM optical sections from the boxed area in ***G***, depicting the cavity in the outlined region. All images are from rat spinal cord 8-9 weeks after AAV injection and spinal cord injury. Scale bars: ***A***, ***G***, 500 μm; ***H***, 200 μm. *n* = 4 biological replicates.

**Figure 7:**
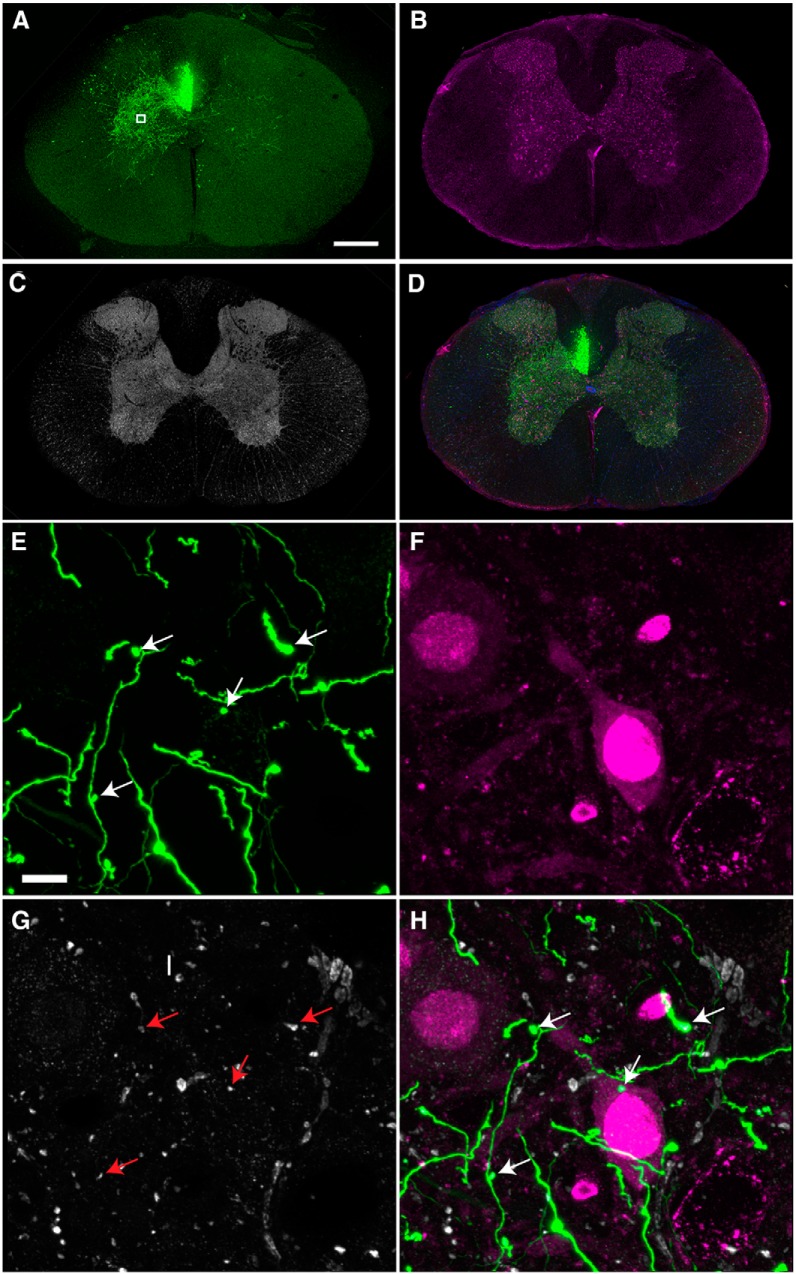
Rat CST terminals labeled with AAV express vGlut1. ***A−D***, Coronal tissue sections of rat thoracic spinal cord immunostained with NeuN in magenta and vGlut1 in gray. GFP-positive CST axons can be seen sprouting into the contralateral and ipsilateral gray matter. ***A***, ***B***, and ***C*** are merged in ***D***. ***E***, ***F***, High magnification view from lamina 6 (boxed region in ***A***) shows colocalization of vGlut1 and GFP terminals (indicated by arrows). All images are from tissue sections from rat spinal cord. Scale bars: ***A−D***, 400 μm; ***E***−***H***, 5 μm. *n* = 3 biological replicates.

While mice offer advantages of being genetically tractable, rats are still widely used across many neuroscience disciplines, including spinal cord injury, since rats (in contrast to mice) display histopathology that is more similar to humans. However, because transgenic labeling of specific subtypes of CNS cells in rats is not widely available, their use has been limited in previous tissue clearing studies (Ertürk et al., 2012b). To determine if virus-based tracing methods can be combined with tissue clearing to study axon trajectory in injured rat spinal cord, we injected AAV8-UbC-GFP into the motor cortex and performed a T8 contusion. In tissue sections from spinal cord segments rostral to the injury site (8-9 weeks after SCI), GFP-labeled axons were observed in all the expected CST tracts (Fig. 6*A−E*). Since pyramidal cells express vesicular glutamate transporter 1 (vGluT1) (Fremeau et al., 2001), we used this as a marker of CST synaptic terminals in the rat spinal cord and found that some of the vGluT1 synaptic terminals in lamina VI colocalize with GFP (Fig. 7) (Persson et al., 2006). It is important to note that all immunohistochemical analyses in our studies are from tissue sections taken from regions adjacent to cleared tissue rather than direct immunostaining of intact cleared tissue as recently reported (Renier et al., 2014). Taken together, our data show that AAV8-UbC-GFP can be used to trace CST axons and their synaptic terminals in the rat spinal cord.

Movie 6 3D LSFM movie of a midthoracic injured rat spinal cord showing the relationship between the lesioned CST labeled with AAV8-UbC-GFP and the cavity highlighted in magenta. Corresponds to Figure 6*F*. 10.1523/ENEURO.0001-15.2015.video.6

Next, we used LSFM to image the injured rat spinal cord after tissue clearing. LSFM optical sections were used to trace and reconstruct the cavities at the injury site (Fig. 5*F*,*G*, Movie 6). Axon terminals and retraction bulbs from the main CST were observed just rostral to the cavity, while dorsolateral CST axons were observed passing by the side of the cavity, suggesting that they were spared, as expected. Therefore, our data show that virus-based tract-tracing can be combined with THF/BABB-based tissue clearing to investigate axon trajectory in the injured spinal cord of nontransgenic animals such as rats.

### Proprioceptive sensory axon projections in the injured rat spinal cord

Dorsal column sensory axons have been studied extensively in terms of their regenerative potential as well as their contribution to local reflexes in various types of SCI models. These axons are typically labeled by direct injection of transganglionic tracers such as CTB into the sciatic nerve or biotinylated dextran conjugates directly into the DRG. However, these injections often result in tissue damage that produces a conditioning effect, which alters gene expression in DRGs and can confound data interpretation. Furthermore, in experiments where bulk labeling of sensory axons are desired (e.g., to study sensory axons along the entire rostro-caudal axis), these traditional labeling methods are not very practical.

**Figure 8:**
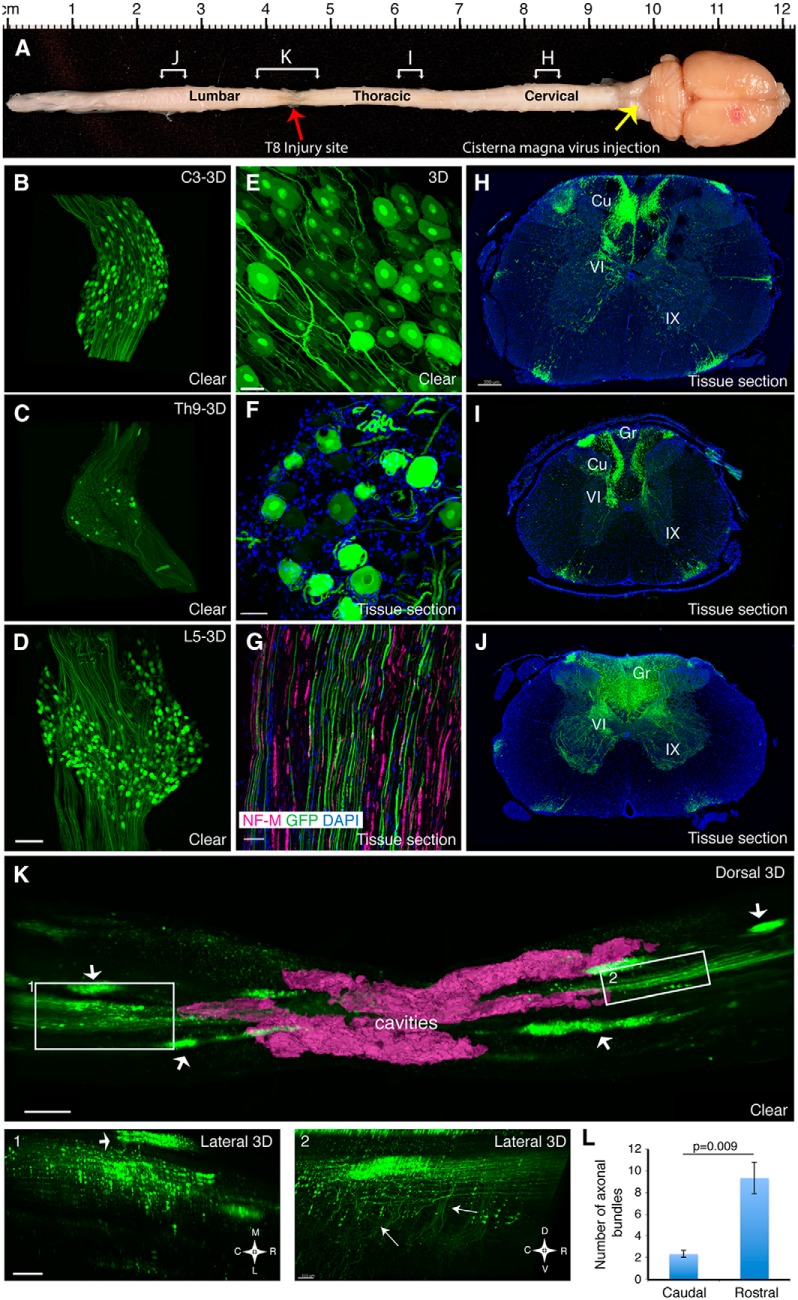
AAV-labeled Ia afferent axons after SCI in rats. ***A***, Adult rat CNS with injured spinal cord at T8 (red arrow) showing the site of virus injection (yellow arrow) and the regions from where the images in ***H−K*** were taken (brackets with corresponding letters). ***B−D***, 3D reconstruction of confocal images from cleared DRG at C3, T9, and L5 levels labelled with AAV8-UbC-GFP. Confocal images of labeled neurons and their axon projections show similar quality between whole cleared DRG (***E***) and DRG tissue sections (***F***). Tissue sections from the cervical (***H***), thoracic (***I***), and lumbar (***J***) spinal cord as well from L5 nerve (***G***) show that this method labels both the central and peripheral branches of the DRG (see also Movie 7). 3D reconstruction of LSFM images from the cleared injury site (***K***, dorsal view) shows the caudal (left, box 1) and rostral (right, box 2) portions of the lesioned ascending sensory axons disrupted by the injury site as outlined by the cavity (magenta, see also Movie 8). Arrows in ***K*** indicate dorsal roots that are still attached to the spinal cord. Arrows in inset 2 indicate afferent collaterals that are present in greater numbers in the rostral regions compared to caudal regions (inset 1). This is quantified in ***L***, which displays the number of axon bundles emanating from the dorsal columns in a similar region of interest drawn rostral and caudal to the injury site in 3D reconstructions. Error bars represent SEM. *p* = 0.009 using unpaired two-tailed Student’s *t* test. All images are from rat spinal cord 8-9 weeks after AAV injection and SCI. Scale bars: ***B−D***, 200 μm; ***E***, 40 μm; ***F***, 50 μm; ***G***, 10 μm; ***H−J***, 300 μm; ***K***, 500 μm; Insets 1, 2, 100 μm. Gr, gracile; Cu, cuneate; VI and IX indicate the Rexed laminae.

**Figure 9:**
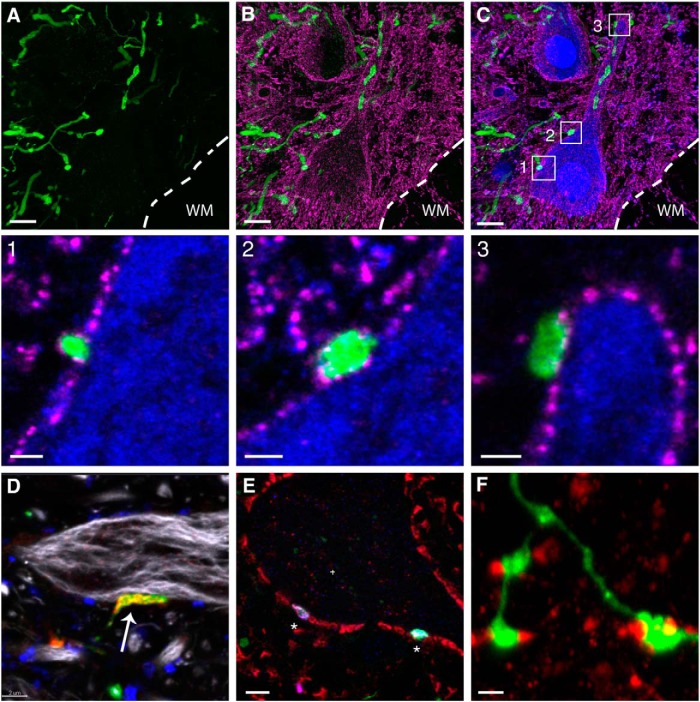
AAV-labeled Ia afferent terminals in rat spinal cord gray matter express vGlut1. ***A−C***, Confocal images of rat spinal cord tissue sections (lamina IX) with GFP-labeled Ia afferents (***A***) immunostained with NeuN (blue) and the presynaptic marker bassoon (magenta). ***C*** is the merged image of ***A*** and ***B***. Insets 1-3 are high-magnification images of corresponding regions depicted in ***C***. ***D***, GFP terminals adjacent to NF-M-positive (gray) axon express vGlut1 (red) but not vGlut2 (blue), indicating their sensory origin. ***E***, GFP terminals express the presynaptic marker SV2 (red) and vGlut1 (blue), indicating their proprioceptive origin. Left asterisk denotes overlapping SV2 and vGlut1, while right asterisk has SV2, vGlut1, and GFP overlapping. ***F***, GAD65 boutons make contacts with GFP terminals, indicating axo-axonic contacts from interneurons onto sensory axons. All images are confocal images of rat spinal cord tissues sections 8-9 weeks after AAV8-UbC-GFP injection. Scale bars: ***A*−*C***, ***E***, 10 μm; Insets 1-3, 1 μm; ***D***, 2 μm; ***F***, 3 μm. *n* = 4 biological replicates.

To address these technical issues, we delivered AAV8-UbC-GFP into the CSF through the cisterna magna in rats and performed T8 contusive SCI in the same animal. 3D reconstructions from confocal images of cleared whole DRGs showed multiple neurons and their processes labeled with GFP (Fig. 8*B−E*, Movie 7). Furthermore, tissue sections of the L5 nerve showed that some large caliber axons were labeled with GFP, indicating that both the central and peripheral branches of the DRG were labeled with this method (Fig. 8*G*). Tissue sections in spinal cord regions distal to the injury site showed a majority of the afferents projecting to lamina VI and the ventral horn, suggesting that most of the GFP-labeled axons were Ia and Ib afferents (Figs. 8*H*,*I*, 9). Fifty-three percent of vGluT1 terminals on the cell bodies and proximal dendrites of L4-L5 motoneurons were colabeled with GFP (Fig. 9*A−D*), suggesting that a large portion of proprioceptive sensory axons were labeled using this method. In addition, we immunostained for several presynaptic markers, including vGlut2, bassoon, SV2, and GAD65. Bassoon immunoreactivity surrounds the NeuN-positive surface and some of them surround the GFP terminals, suggesting the presence of axoaxonic synapses on the GFP terminals (Fig. 9*A−C* and insets 1-3). While none of the GFP terminals were vGlut2-positive (Fig. 9*D*), all expressed the presynaptic marker SV2 (Fig. 9*E*). We also found that GABAergic (GAD65-positive) boutons surround GFP terminals (Fig. 9*F*). Taken together, our results indicate that the GFP boutons are of sensory origin. However, it should be noted that ∼40% of the AAV injected animals expressed GFP in other white-matter tracts (Fig. 8*H−J*), most likely due to labeling of neurons that give origin to descending tracts located close to the cisterna magna (data not shown).

Movie 7 3D LSFM movie showing cleared L5 adult rat DRG and L2 rat spinal cord segment (caudal to a midthoracic injury) in which sensory neurons and afferents are labeled after injection of AAV8-UbC-GFP into the cisterna magna. Corresponds to Figure 8*D*. 10.1523/ENEURO.0001-15.2015.video.7

3D reconstructions of the injury site clearly showed the cavity boundaries and the injured axons both caudal and rostral to the injury site (Fig. 8*K*). Dorsal column axons caudal to the injury were interrupted and displayed numerous retraction bulbs and varicosities with a notable absence of Ia afferent collaterals in the spinal gray matter (Fig. 8, inset 1, Movie 8). While ascending axons rostral to the injury also displayed varicosities and retraction bulbs, axon collaterals into the gray matter were observed close to the cavities (Fig. 8*K*, inset 2, Movie 8). Taken together, our data demonstrate an alternative method of labeling ascending dorsal column sensory axons using AAV virus that can be used to visualize sensory axons in cleared rat whole spinal cord to assess axonal response after SCI.

Movie 8 3D LSFM movie showing lesioned rat sensory fibers labeled with AAV8-UbC-GFP and the cavity (highlighted in magenta) around a midthoracic injury site. Corresponds to Figure 8*K*. 3D reconstructions of insets 1 and 2 are also included to demonstrate the lack of afferent collaterals in the distal segment (inset 1) in contrast to their presence in the proximal segment (inset 2). 10.1523/ENEURO.0001-15.2015.video.8

**Figure 10:**
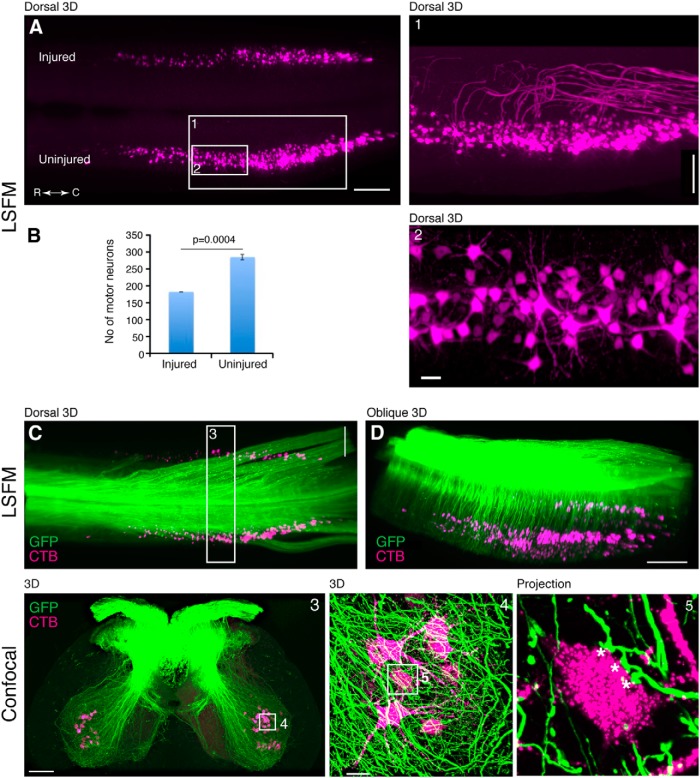
AAV-labeled Ia sensory afferents innervating CTB-labeled rat gastrocnemius motor pool targets. ***A***, 3D reconstruction of LSFM images from rat lumbar spinal cord with gastrocnemius motor pool retrogradely labeled with CTB. Inset 1 is a magnified view with the motor axons highlighted using a filtering mask. Inset 2 is a magnified view showing the motoneuron soma and dendrites. The difference in motoneuron number between the two sides is due to the unilateral lumbar SCI is quantified in ***B***. ***C***, ***D***, 3D reconstruction in ***A*** shown together with GFP-labeled sensory axons (also see Movie 9). Inset 3, Confocal transverse images of a cleared spinal cord segment outlined in ***C***. Inset 4 depicts a network of axons and their terminals surrounding CTB-labeled motoneurons. Inset 5 is a projection of 15 confocal optical sections showing three “en passant” boutons (asterisks) on a single motoneuron (see also Movie 10). All cords were examined 8-9 weeks after the AAV injection. All images are from cleared spinal cord from the same animal. Scale bars: ***A***, 500 μm; ***C***, 500 μm; ***D***, 700 μm; Inset 1, 300 μm; Inset 2, 50 μm; Inset 3, 200 μm; Inset 4, 30 μm. *n* = 3 biological replicates.

In addition to projecting to the gracile and cuneate nuclei in the brainstem, ascending proprioceptive sensory axons also send collaterals that synapse directly onto motoneurons as part of the monosynaptic reflex. Since these local connections are affected by SCI both anatomically and physiologically (Lee et al., 2007; Rotterman et al., 2014), we used this as a model system to study target innervation by labeled axons using our tissue clearing approach. Furthermore, in order to visualize how motor pools are directly affected by SCI, we used an unilateral lumbar (L5) contusion model to measure the death of motoneurons, which were retrogradely labeled by injecting CTB conjugated to Alexa Fluor 555 into the LG and MG muscles. The entire motor pools were observed in 3D using LSFM, and their cylindrical and longitudinally oriented columns were consistent with earlier reconstructions using histological sections of horseradish peroxidase-labeled motoneurons (Fig. 10*A*, Movie 9) (Swett et al., 1986). In the uninjured side, the total number of LG and MG motoneurons was 287 ± 14, which is similar to previous reports (Swett et al., 1986). In the injured side, the number of motoneurons was 183 ± 9, indicating that our 3D analysis was sensitive enough to detect an expected decrease in motoneurons after lumbar SCI (Fig. 10*B*).

Movie 9 3D LSFM movie of rat lumbar spinal cord after a unilateral lumbar injury. Gastrocnemius motor pools were retrogradely labeled using CTB-Alexa 555 (GM-CTB, shown in purple) and sensory axons were labeled by injecting AAV8-UbC-GFP into the cisterna magna (sensory-GFP, shown in green). Later movie segments show motor axons that were traced manually using Imaris. Corresponds to Figure 10*A−E*. 10.1523/ENEURO.0001-15.2015.video.9

Next, we sought to determine the spatial distribution of the sensory axon contacts onto CTB-labeled motoneurons. Using LSFM, we observed axon bundles as well as single axons entering the spinal cord and projecting into the ventral gray matter near the vicinity of the retrogradely labeled motoneurons (Fig. 10*C*,*D*, Movie 9). Since LSFM was insufficient to resolve terminal contacts onto motoneurons, we cut 300-400 μm pieces of the same spinal cord and imaged with a confocal microscope. Using a 60× objective (NA 1.42), we were able to observe terminal contacts from proprioceptive sensory axons onto individual motoneurons as well their dendrites (Fig. 10*C*, insets 3, 4, 5, Movie 10). The 3D reconstruction shows the gray matter overabundant with GFP fibers, making it difficult to distinguish individual fibers and terminals unless a few optical sections were viewed in 2D (Fig. 10*C*, inset 4, Movie 10). However, 3D reconstructions combined with volume rendering of specific motoneurons revealed GFP terminals more clearly (Movie 10). Furthermore, projection of small numbers of optical sections can also differentiate terminals ending on a neuron from fibers passing nearby but in a different plane (Fig. 10*C*, inset 5). Therefore, our approach can be used to investigate axonal innervation of target neurons in rat whole spinal cord without the need for transgenic labeling.

Movie 10 3D confocal movie of gastrocnemius motoneurons labeled with CTB-Alexa 555 and sensory fiber terminals labeled with AAV8-UbC-GFP in the cleared rat lumbar spinal cord (as shown in Figure 10, inset 4). First segment of the movie shows all the *z*-stack images taken from the cleared tissue using confocal microscopy. Next segment shows a 3D reconstruction of these *z*-stacks. This is followed by manual rendering of individual motoneurons (from this 3D reconstruction) with synaptic contacts (red dots) from labeled afferent axons (yellow). 10.1523/ENEURO.0001-15.2015.video.10

### CST axon projections in the nonhuman primate spinal cord

**Figure 11:**
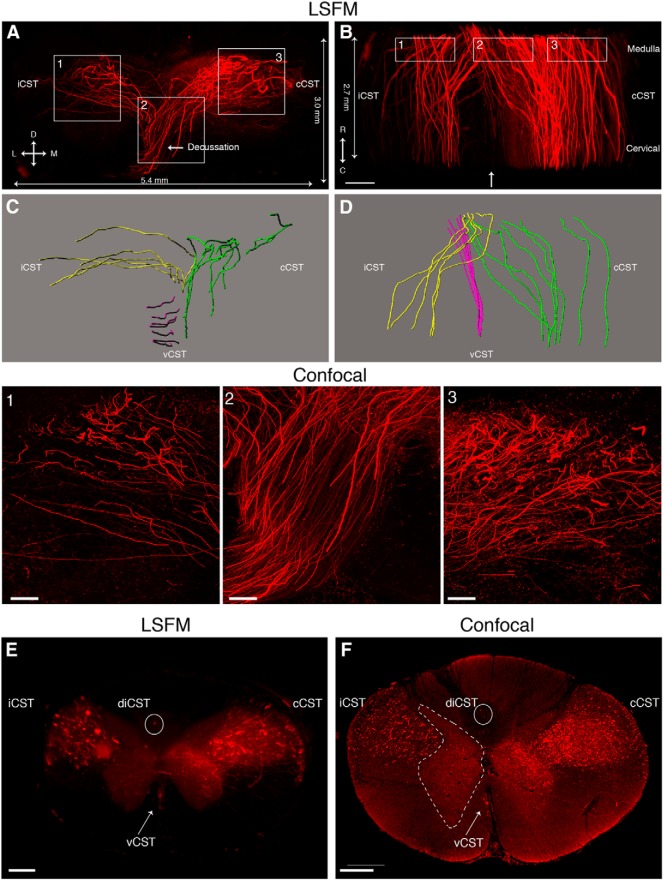
3D visualization of fluoro-Ruby-labeled CST axons in cleared nonhuman primate spinal cord. **A**, ***B***, 3D reconstruction of LSFM images showing fluoro-Ruby-labeled CST axons at the level of the pyramidal decussation in cleared nonhuman primate tissue (see also Movie 11). Lines on the edges indicate the length of the cleared specimen imaged by LSFM. Actual thickness of the cleared tissue was 5 mm. Insets 1, 2, and 3 are confocal images of the same cleared tissue taken from corresponding regions depicted in ***A*** and ***B***. The confocal scans are ∼400 μm in depth and taken from the rostral side of the tissue. ***C***, ***D***, Axon tracings from ***A*** and ***B***, respectively, showing the iCST (ipsilateral CST, yellow), cCST (contralateral CST, green), and vCST (ventral CST, magenta). Images are slightly tilted to reveal the projections better and do not represent the exact orientation of images in ***A*** and ***B***. ***E***, ***F***, Coronal views from LSFM (C2 level in ***E***) and confocal (C1 level in ***F***) images show the main contralateral (cCST) and ipsilateral (iCST) CST in the dorsolateral white matter as well as the minor ventral CST (vCST, arrow) in the ventromedial white matter. Note the presence of two CST axons in the ipsilateral dorsal column (diCST, circle). The gray matter in ***F*** is delineated with dashed lines. All images are from cleared nonhuman primate tissue injected with fluoro-Ruby. L, Lateral; M, medial; D, dorsal; V, ventral; R, rostral; C, caudal. Scale bars: ***A***, ***B***, ***E***, ***F***, 600 μm; insets 1-3, 150 μm. *n* = 1.

Due to differences between rodents and primates in the organization of motor pathways, as well as immune and other pathophysiological responses after SCI, nonhuman primate models have an important role in SCI research. To determine if supraspinal axons can be imaged in cleared nonhuman primate spinal cord, we stereotacticaly injected fluoro-Ruby into the right primary motor cortex in a macaque. Ten weeks later, the animal was euthanized and two spinal cord regions were cleared; the first was a 5 mm piece at the boundary of the medulla and the cervical spinal cord and the other piece was at C2 (Fig. 11). In the first piece (medulla-cervical border), CST axons located in the pyramidal decussation were observed descending from the ventral pyramids into the main lateral CST (Fig. 11*A*,*B*, Movie 11). Few axons were observed traveling in the ipsilateral ventral cord and even fewer were observed in the dorsal columns. While LSFM was able to discern many individual CST fibers that were traceable by Imaris software (Fig. 11*C*,*D*), confocal microscopy allowed the visualization of additional, thinner CST (Fig. 11, insets 1-3). In the cleared C2 segment, most of the axons run parallel to the main axis of the spinal cord with collateral fibers innervating the gray matter (Fig. 11*E*,*F*, Movie 11). This CST organization in macaque is consistent with previous descriptions of the CST in other nonhuman primates (Lacroix et al., 2004). Taken together, these results show that our approach can be used to visualize supraspinal axons in cleared nonhuman primate spinal cord.

Movie 11 3D LSFM movie from cleared nonhuman primate medulla and cervical spinal cord showing the CST labeled with fluoro-Ruby. The first segment of the movie shows CST axons at the level of the pyramidal decussation. The second segment shows CST axons at C2 level of the spinal cord. Corresponds to Figure 11. 10.1523/ENEURO.0001-15.2015.video.11

## Discussion

Although recent advances in tissue-clearing techniques have made it much easier to obtain accurate origin-target information from whole tissue, most previous studies have relied on transgenic mice in which multiple axonal populations are brightly prelabeled with GFP. In this study, we have optimized viral- and chemical-based tract-tracing strategies that can be combined with tissue clearing and imaging techniques to study axonal projections in the mouse, rat, and nonhuman primate spinal cord. This approach provides researchers with better spatiotemporal control over axonal labeling as well as the flexibility to use these methods in various experimental paradigms, including those that require model organisms in which transgenic axonal labeling is unavailable.

By comparing different combinations of AAV serotypes, promoters, and fluorescent proteins, we determined that AAV2 or AAV8 can be used with the UbC promoter to express either GFP or tdTomato. While AAV8-UbC-GFP provided the best results, AAV8 (or 2)-UbC-tdTomato permits axon tracing in transgenic mouse lines, a majority of which express GFP. In addition, the use of GFP and tdTomato simultaneously allows labeling of multiple tracts in the same animal. However, when we injected AAV8-UbC-GFP and AAV8-UbC-tdTomato to label each CST in the same rat, we found that fine collaterals were much better labeled with GFP than tdTomato. This could be due to several potential factors, including increased susceptibility of tdTomato to quenching by THF/BABB, higher expression of GFP compared to tdTomato, better optimization of the translation code in the *GFP* gene, and/or better signal-to-noise ratio in the green channel in our imaging systems.

In addition to the virus serotype and promoter, another important variable is the time allowed after virus injection. In mice, we allowed 4 weeks after virus injection, which in our experience is optimal for achieving sufficient labeling of fine processes in the gray matter. Two weeks is sufficient for histological sections, but for applications using THF/BABB tissue-clearing method, there is significant degradation of endogenous fluorescence in fine processes (our unpublished observations; Table 1). In rats, we allowed 8-9 weeks after virus injection due to their larger size. Four weeks after virus injection in mice (and 8-9 weeks in rats), we were able to image cleared tissue for about 4-5 d before significant quenching. However, chemical-based tracers (such as dextran conjugates) lasted for several months after initial tissue clearing.

**Table 1.** Comparison of fluorescence quenching between different neural labeling methods after 3DISCO (with BABB) tissue clearing

Axonal/neuronal labeling with:	Fluorescence quenching	Ideal imaging time frame	Comments
AAV-eGFP, tdTomato	Fluorescence disappears progressively within a week	Within 48 h	eGFP is preferred over tdTomato
Fluoro-Ruby, Fluoro-Emerald	Fluorescence remains for few months	Within first few weeks	Results in off-target labeling of perivascular macrophages
CTB-conjugated to Alexa fluors	Fluorescence remains for few months	Within first few weeks	Preferentially labels cell body rather than axon; axon visualization requires software filtering
Whole-mount immunofluorescence with Alexa fluors	Fluorescence remains for few months	Within first few weeks	e.g., Whole-mount staining of embryos followed by tissue clearing

Based on results from this study and our unpublished observations.

While viruses expressing fluorescent proteins offer the advantage of continuous expression of the fluorescent marker, certain experiments may require tract-tracing using other types of tracers. For example, for retrograde tracing, the use of fluorophore-conjugated CTB is much more common than the use of retrograde viruses. In our study, we used CTB conjugated to Alexa Fluor 555 to retrogradely label LG and MG motoneurons and studied their direct innervation by proprioceptive sensory axons labeled with AAV8-UbC-GFP, demonstrating that both virus and conventional tract tracing are simultaneously compatible with the THF/BABB tissue-clearing method. In addition, since chemical fluorophores (such as Alexa dyes) are much more resistant to degradation by THF/BABB as compared to fluorescent proteins, they may be more suitable for tract-tracing in larger animals that require longer THF/BABB incubation times. In our study, we used a dextran conjugate (fluoro-Ruby) to label the CST and obtain a 3D reconstruction of CST axons projecting through the cleared macaque spinal cord. To our knowledge, our study is the first to demonstrate axonal projections in cleared spinal cord from a nonhuman primate as well as from rats with spinal cord injury. It is also important to note that clearing adult spinal cord tissue poses special challenges because of the abundance of myelin compared with brain or embryonic tissue, which has often been used to demonstrate tissue-clearing methods in recent studies (Chung et al., 2013; Belle et al., 2014). In addition, while chemical fluorophores are much more resistant to quenching, in our experience, injection of dextran conjugates (such as fluoro-Ruby; Table 1) to label CST in rodents often leads to labeling of perivascular macrophages throughout the CNS.

The ability to clearly demonstrate axonal projections is especially important in axon regeneration studies, which are plagued with the misidentification of spared axons in regions caudal to the injury site as regenerated axons (Tuszynski and Steward, 2012). However, by combining tissue clearing with tract-tracing methods similar to those that are typically used in axon regeneration studies, we were able to identify axons that were mislabeled or present in atypical regions that could be easily misinterpreted as regenerating axons. For example, when we labeled RST axons in mice, we found that the small size of the red nucleus often led to labeling of surrounding nuclei with descending projections in the ventral white matter (Fig. 3*E−G*). In addition, CST tracing in the macaque revealed a few CST axons in the ventral portion of the dorsal columns (Fig. 11*E*,*F*). In both instances, researchers observing these “ectopic” axons in tissue sections could have easily misinterpreted them as having regenerated. However, 3D reconstructions from cleared whole spinal cord convincingly demonstrated the location and trajectory of these axons, and such information can be used to correctly distinguish between axons that have regenerated from those that were spared from injury either due to mislabeling or presence in atypical regions.

Tissue clearing, of course, is only one of several steps required for obtaining axonal projection information from whole tissue. Another important aspect that is often insufficiently addressed is the technical challenges in microscopy required to capture and reconstruct images from whole tissue. We used the ultramicroscope version of LSFM to image large pieces of the spinal cord, which provided rapid image acquisition (∼3 min for ∼6-mm-long spinal cord segment) and gross anatomical information on axonal projections. For large diameter axons such as in the RST and ascending sensory axons, we were able to observe individual axons using LSFM. However, for thinner axons such as CST axons, or for fine anatomical structures such as synaptic terminals, confocal microscopy was necessary to provide the required resolution. Since it is impractical to acquire images from the entire spinal cord using confocal microscopy, we focused on specific areas with confocal microscopy after using LSFM. This required us to cut the cleared tissue into smaller segments to make it compatible with the working distances of our confocal objectives. While we could have used a two-photon microscope to acquire similar high-resolution images without having to cut the cleared tissue into smaller segments, confocal microscopy is a more practical approach since it is much more readily available to the general scientific community.

It is important to note that we used the La Vision BioTec Ultramicroscope, which was designed to image large tissue such as rodent brain by using a fluorescence macro zoom microscope (Olympus MVX10) with a 2× objective and a total magnification of 12.5×. As demonstrated by our images of fine CST axons, these specifications sacrifice resolution for large sample size and acquisition speed. However, it is possible that other LSFMs with higher power objectives such as SPIM (Selective Plain Illumination Microscopy) or ezDSLM (easy Digital-Scanned Light Sheet Microscopy) may provide better resolution of fine axons (Huisken et al., 2004; Takao et al., 2012). In fact, SPIM was recently used to develop COLM (CLARITY-optimized light sheet microscopy) using a 10× (NA 0.60) objective, which should have slightly better resolution than our Ultramicroscope (Tomer et al., 2014). However, it takes hours to image a whole mouse brain using COLM, whereas the Ultramicroscope can do it in minutes. Therefore, the user must consider the advantages and disadvantages of each method and choose the one that best suits their experimental needs.

In summary, we present optimized and comprehensive methods for using tract-tracing to investigate axonal projections in cleared whole mouse, rat, and nonhuman primate spinal cords. While we focused on the injured spinal cord, this procedure can readily be applied to uninjured brain and spinal cord in connectomic studies. In addition, our methods can be combined with existing transgenic mouse lines or used in species in which transgenic labeling is unavailable, providing researchers with enhanced flexibility in investigating axonal trajectories in multiple species with improved accuracy and speed.

## Synthesis

The decision was a result of the Reviewing Editor Michael Sofroniew and the peer reviewers coming together and discussing their recommendations until a consensus was reached. A fact-based synthesis statement explaining their decision and outlining what is needed to prepare a revision is listed below. The following reviewer agreed to reveal his identity: Binhai Zheng

Reviewer #1 comments:

In this work, the authors demonstrated new applications of THF-BABB based tissue clearing to study spinal cord injury. While recently developed tissue clearing methods opened the way to study neuronal connections in intact spinal cords, labeling the tissue of interest remains as the major obstacle. Hence, the authors' sophisticated demonstration of the axonal tracts in the spinal cord using AVVs and tracers will allow labeling and imaging of non-transgenic animals for SCI studies, which mostly use rats rather than reporter mice.

My major comment on the paper is the necessity of some quantifications of observed neuronal structures to prove this method is not merely to take qualitative images and videos. It is clear also in their findings that one can software-trace and quantify the axons or neurons in the spinal cord. It would be also valuable demonstrate this here. Please see more specific comments figure by figure below.

Figure 1:

Authors indicate that AVV8 -UbC shows the best labeling. Please provide quantitation (e.g. fluorescent intensity and axon numbers) for different labeling methods to statistically claim that this is the case.

Please label figures columns for color codes, DAPI etc.

Figure 2:

Figure 2A and 2B looks very flat (2D) unlike the video file. Please improve it to demonstrate that what we are looking at is really a 3D image. What is the minimum time needed to get a good AVV labeling? Have you tried 1 or 2 weeks? Please at least mention this in the discussion with previous literature.

Please indicate the length of the spinal cord imaged within the figure. Imaging several millimeters of the spinal cord is alone an achievement and should be made clear to readers.

What are the post-injury times, please indicate at each figure whenever applies.

Figure 4:

Can you please provide quantification to compare the clearing efficiency between light-sheet and confocal imaging for the reporter mice?

Figure 5:

Can you please software-trace some of these axons in the rats? For example, some axons look uncut. Can you trace the spared axons versus the cut ones to show that the technique (3D imaging of traced axons after tissue clearing) can differentiate between spared vs. degenerating/regenerating axons?

Figure 7:

Authors write "While ascending axons rostral to the injury also displayed varicosities and retraction bulbs, axon collaterals into the gray matter". Please quantify and present the data to make a statistical point for this result.

Figure 9:

Can you please make a graph for quantification in the figure with a suitable statistical test?

Figure 10:

The authors demonstrate fluororuby-traced axons in monkey. This looks impressive. Can you please software trace some sample axons?

ADDITIONAL points:

Please label all figures (e.g. Figure 1) for reviewers. It is hard to read the paper without promptly knowing which figure is which.

Please label in all figures what is cleared tissue, maybe with a color or shape code at each corner.

Please indicate in figures which samples are injured and at which side they are injured if a unilateral lesion was applied e.g. in Figure 9A.

Please label the complete size of large tissues that are imaged in the figures as well. For example Fig. 10.

Can you please make a few simple drawings to demonstrate how the labeling is achieved (in which region is the virus/tracer injected), for example in Figure 7.

Reviewer #2 comments:

This manuscript titled "3D imaging of axons in transparent spinal cords from rodents and non-human primates" is a proof-of-concept study describing AAV or chemical (Alexa-coupled Cholera Toxin B subunit, Fluoro-Ruby) -based tracing of axonal fibers combined with three dimensional imaging of THF/BABB cleared tissue. Whereas other studies were limited by using transgenic mice in combination with tissue clearing and 3D imaging, the current manuscript describes 3D imaging of spatiotemporal controllable labeling of axons in mice, rats and non-human primates. The authors elegantly show feasibility as well as limitations of the clearing protocol and the available 3D imaging techniques by combining viral-based tracing with transgenic labeling in mice or chemical-based tracing in rats. Fluoro-Ruby labeling of CST axons allows for 3D imaging at single axon resolution in cleared Macaque spinal cord.

This manuscript represents an important improvement in methodology, which for the first time illustrates three dimensional imaging of surgically traced axons in uninjured and injured spinal cord in rodents and non-human primates. However, the presentation of the Results and the Discussion can be improved in several aspects.

1) Further clarifying the relationship between videos and figures (in legends) would help. State clearly which species was used in each figure/video.

2) Scale Bars are sometimes hard to see (Figures 5G, 8D, 9B2 and confocal images) or missing (Figures 2E-H, 3D-J). Figure 5 legend refers to a scale bar for a non-existing panel L.

3) Clearly state where sections came from in comparison to the cleared tissue (Lines 379 - 382). The authors should also make it clear that antibody staining was not performed in combination with tissue clearing, such as with the recently published iDISCO method (Renier et al., Cell. 2014 Nov 6;159(4):896-910. doi: 10.1016/j.cell.2014.10.010.)

4) The authors state that tdTomato can be used for axon tracing (Line 511-514, Figure 3). A direct comparison of the AAV8-UbC-GFP and the AAV8-UbC-tdTomato would help to show that single tdTomato labeled axons can be imaged after using the modified 3DISCO clearing method. TdTomato and eGFP have previously been reported to be rapidly quenched by some tissue clearing techniques (Ke et al., 2013, Nature Neuroscience 16, 1154-1161 (2013) doi:10.1038/nn.3447; Renier et al., 2014). Results presented in Figure 3 also suggest that tdTomato is quenched more rapidly than eGFP. A direct comparison of sections and cleared tissue labeled with tdTomato and eGFP on either hemisphere would further clarify this issue. In this context: Can eGFP or tdTomato labeling be used in non-human primate tissue? If the authors have done this, please include some examples. If the longer incubation time in THF results in quenching of the fluorescence signals this should be discussed.

5) The authors state that light sheet microscopy cannot be used to image at high resolution (line 704). However, specifics concerning the objectives used are given for confocal microscopy but not for light sheet microscopy (line 245). The authors may want to discuss their results in the context of the COLM technique (Tomer et al 2014; Nat Protoc. 2014 Jul;9(7):1682-97. doi: 10.1038/nprot.2014.123). This CLARITY optimized light sheet microscopy technique has been reported to combine high resolution and rapid 3D imaging.

6) What are the reasons for the limited imaging depth using confocal microscopy (line 732)? Is this caused by limited working distance of the objective or light scattering in the cleared tissue? Would 2-photon microscopy allow for deeper penetration of the tissue while maintaining high resolution?

7) For Imaris software analyses, please provide a bit more details how this was done, e.g. were the axons automatically traced or semi-automatic?

Minor:

Check consistency in wording (e.g. labeling vs labeling; Figure vs Fig).

## Author Response

We thank the reviewers for their generous comments about our work and the constructive criticisms for improving the manuscript.

We have made changes based on the reviewers’ comments. Please see below for our point-by-point response. In response to reviewer 2’s suggestion, we have included a new figure showing differences between GFP and tdTomato labeling. This data is now Figure 2, which has resulted in all the ensuing figures increasing by one. Reviewers’ comments refer to the old figure number and we have attempted to refer the reviewers to the new figure number where appropriate. Revised text in the manuscript is denoted in blue.

**Reviewer 1 comment:**

Figure 1:

Authors indicate that AVV8 -UbC shows the best labeling. Please provide quantitation (e.g. fluorescent intensity and axon numbers) for different labeling methods to statistically claim that this is the case.

Please label figures columns for color codes, DAPI etc.

**Response:** We provide quantification of the fibers in the gray matter comparing the three viruses. We have also included color codes at the top of each column.

**Reviewer 1 comment:**

Figure 2 (now Figure 3):

Figure 2A and 2B looks very flat (2D) unlike the video file. Please improve it to demonstrate that what we are looking at is really a 3D image. What is the minimum time needed to get a good AVV labeling? Have you tried 1 or 2 weeks? Please at least mention this in the discussion with previous literature.

Please indicate the length of the spinal cord imaged within the figure. Imaging several millimeters of the spinal cord is alone an achievement and should be made clear to readers.

What are the post-injury times, please indicate at each figure whenever applies.

**Response:** We have modified the images to make it more 3D. In our experience the optimal labeling time for tissue clearing in mice is 4 weeks. Two weeks is sufficient for histological analysis, but we recommend 4 weeks in order to achieve sufficient GFP labeling in fine CST fibers. We have included this in our discussion

We have included post-injury times in all figure legends where appropriate.

**Reviewer 1 comment:**

Figure 4 (now Figure 5):

Can you please provide quantification to compare the clearing efficiency between lightsheet and confocal imaging for the reporter mice?

**Response:** We have performed the quantification by counting the number of axons that can be accurately traced in each condition using the Imaris software. We found that confocal imaging leads to about 50% more axons that can be counted as compared to LSFM. We have included this quantification in the figure and the corresponding text.

**Reviewer 1 comment:**

Figure 5 (now Figure 6):

Can you please software-trace some of these axons in the rats? For example, some axons look uncut. Can you trace the spared axons versus the cut ones to show that the technique (3D imaging of traced axons after tissue clearing) can differentiate between spared vs. degenerating/regenerating axons?

**Response:** We have traced the CST axons and included the new images in our figure. We traced the spared axons in the dorsolateral CST as well as the severed axons next to the cavity and added the new images in the figure.

**Reviewer 1 comment:**

Figure 7 (now Figure 8):

Authors write "While ascending axons rostral to the injury also displayed varicosities and retraction bulbs, axon collaterals into the gray matter". Please quantify and present the data to make a statistical point for this result.

**Response:** We have performed the quantification and included it in our revised manuscript.

**Reviewer comment:**

Figure 9 (now Figure 10):

Can you please make a graph for quantification in the figure with a suitable statistical test?

**Response:** We have included a bar graph with a suitable statistical test in the figure.

**Reviewer 1 comment:**

Figure 10 (now Figure 11):

The authors demonstrate fluororuby-traced axons in monkey. This looks impressive. Can you please software trace some sample axons?

**Response:**

We have performed tracing using the Imaris software and have included the images in the figure.

ADDITIONAL points from reviewer 1:

Please label all figures (e.g. Figure 1) for reviewers. It is hard to read the paper without promptly knowing which figure is which.

**Response:** We have labeled each figure.

Please label in all figures what is cleared tissue, maybe with a color or shape code at each corner.

**Response:** We have now clearly stated this either in the figure panels or in the figure legends for each figure.

Please indicate in figures which samples are injured and at which side they are injured if a unilateral lesion was applied e.g. in Figure 9A.

**Response:** We have corrected this as suggested by the reviewer.

Please label the complete size of large tissues that are imaged in the figures as well. For example Fig. 10 (now Figure 11).

**Response:** We have included the tissue size in the figure panels where appropriate. Can you please make a few simple drawings to demonstrate how the labeling is achieved (in which region is the virus/tracer injected), for example in Figure 7 (now Figure 8).

**Response:** We have added a diagram for clarification

**Reviewer 2 comment:**

1) Further clarifying the relationship between videos and figures (in legends) would help. State clearly which species was used in each figure/video.

**Response:** We have revised the legends to make the figure-video relationship clearer and to include the species.

**Reviewer 2 comment:**

2) Scale Bars are sometimes hard to see (Figures 5G, 8D, **9B2** and confocal images) or missing (Figures 2E-H, 3D-J). Figure 5 legend refers to a scale bar for a non-existing panel L. (All figure numbers are now increased by one)

**Response**: We thank the reviewer for catching some of the missed scale bars. We have corrected the scale bars and the reference to panel L.

**Reviewer 2 comment:**

3) Clearly state where sections came from in comparison to the cleared tissue (Lines 379 - 382). The authors should also make it clear that antibody staining was not performed in combination with tissue clearing, such as with the recently published iDISCO method (Renier et al., Cell. 2014 Nov 6;159(4):896-910. doi: 10.1016/j.cell.2014.10.010.).

**Response:** We have clarified this and cited the paper referenced by the reviewer.

**Reviewer 2 comment:**

4) The authors state that tdTomato can be used for axon tracing (Line 511-514, Figure 3) (now Figure 4). A direct comparison of the AAV8-UbC-GFP and the AAV8-UbCtdTomato would help to show that single tdTomato labeled axons can be imaged after using the modified 3DISCO clearing method. TdTomato and eGFP have previously been reported to be rapidly quenched by some tissue clearing techniques (Ke et al., 2013, Nature Neuroscience 16, 1154-1161 (2013) doi:10.1038/nn.3447; Renier et al., 2014). Results presented in Figure 3 (now Figure 4) also suggest that tdTomato is quenched more rapidly than eGFP. A direct comparison of sections and cleared tissue labeled with tdTomato and eGFP on either hemisphere would further clarify this issue. In this context: Can eGFP or tdTomato labeling be used in non-human primate tissue? If the authors have done this, please include some examples. If the longer incubation time in THF results in quenching of the fluorescence signals this should be discussed.

**Response:**

To address the reviewer’s comments regarding comparison of GFP and tdTomato, we have provided additional data (new Figure 2) where we labeled each CST with either GFP or tdTomato virus in the same rat. Based on our results, GFP seems to be the better fluorescent protein in labeling CST axons. As the reviewer suggested, one possible reason for the worse labeling observed with tdTomato is its higher susceptibility to quenching after THF/BABB incubation. However, there are other reasons that need to be considered. Based on our observations, GFP seems to be expressed at higher levels than tdTomato. This could be due, in part, to the size of the genes; Tdtomato is twice the size of GFP. In addition, expression levels also depend on the optimization of translation code. In preliminary studies, we used two different tdTomato genes. The one described in the paper was made synthetically and optimized to the human codon usage. This expresses better than the one we originally obtained from Addgene. Even with this improvement, GFP seems to be expressed at higher levels. In addition, differences in signal-to-noise ratio between the green and red channels, which can vary from one confocal system to another, could also have been a contributing factor. We have included these possibilities in our discussion. As for the “rapidly” quenching mentioned in some of the papers the reviewer mentions above, this is partially true. However we are able to image samples 4-5 days after the initial clearing, so the quenching is probably not as rapid as claimed in some of the manuscripts. In our hands the alexa fluors or dextran-conjugates like the ones used in our studies maintain their fluorescent intensity for several months. We have included this in our discussion.

We have not used our viruses in non-human primates yet, but we are planning to do so in the near future.

**Reviewer 2 comment:**

5) The authors state that light sheet microscopy cannot be used to image at high resolution (line 704). However, specifics concerning the objectives used are given for confocal microscopy but not for light sheet microscopy (line 245). The authors may want to discuss their results in the context of the COLM technique (Tomer et al 2014; Nat Protoc. 2014 Jul;9(7):1682-97. doi: 10.1038/nprot.2014.123). This CLARITY optimized light sheet microscopy technique has been reported to combine high resolution and rapid 3D imaging.

**Response:**

We have now included the specifications for the LSFM objectives and have included a discussion comparing COLM to our method as suggested by the reviewer. There are several versions of LSFMs and some can image at relatively high resolutions as initially demonstrated using SPIM by Stelzer’s group (Huisken et al 2004; *Science* **305** (5686): 1007–1009. doi:10.1126/science.1100035. PMID 15310904.) or with ezDSLM that uses a 40x objective with a NA of 0.80 (Takao et al PLoS ONE 7(12): e50846. doi:10.1371/journal.pone.0050846). The resolution in any LSFM setup is determined by the objective’s N.A and the camera. We used the ultramicroscope version of LSFM, which is based on a fluorescence macro zoom microscope (MVX10, Olympus) with a 2x Plan Apochromat zoom objective (NA=0.50) and the capability of zooming up to 12.5x. The ultramicroscope was designed with the sole purpose of imaging centimeter-sized specimens, such as the rodent brain, albeit at the cost of sacrificing resolution. The COLM uses the SPIM version of LSFM in combination with CLARITY tissue clearing technique. COLM utilizes the new high NA 10x objective from Olympus, which in theory, would provide slightly better resolution than our ultramicroscope. However, it takes 4 hours to image an entire mouse brain because it requires multi-tiling. With our ultramicroscope, we can image the whole mouse brain at 2-3x in about 5 minutes without multi-tiling. At the highest magnification of 12.5x, which covers about a quarter of the mouse brain, we can image the entire depth of the brain in about 5-10 minutes. Therefore, while our method has slightly lower resolution compared to COLM, our image acquisition is at least an order of magnitude faster than COLM.

**Reviewer 2 comment:**

6) What are the reasons for the limited imaging depth using confocal microscopy (line 732)? Is this caused by limited working distance of the objective or light scattering in the cleared tissue? Would 2-photon microscopy allow for deeper penetration of the tissue while maintaining high resolution?

**Response:**

Yes, the limited imaging depth using our confocal was mostly due to the working distance of our objectives. Unfortunately, we have an older confocal microscope from Olympus (FV1000) that accommodates objectives with a 25 mm nosepiece thread. The new long working distance 10-25x objectives from Olympus with high NA, like the ones used in the COLM paper, are designed with a 32 mm nosepiece thread. This new design is essential to obtain the high NA. These objectives are not compatible our microscope and therefore we were not able to use them to image deeper. Using a 2- photon microscope would circumvent this problem and we had mentioned this in our discussion (lines 560-565 in original submission)

**Reviewer 2 comment:**

7) For Imaris software analyses, please provide a bit more details how this was done, e.g. were the axons automatically traced or semi-automatic?

**Response:**

We have provided more detail in our methods.

Minor:

Check consistency in wording (e.g. labeling vs labeling; Figure vs Fig).

We thank the reviewer for pointing this out to us. We have corrected the inconsistencies in our manuscript.
